# Enhanced transdermal delivery of pioglitazone hydrochloride via conductive hydrogel microneedles combined with iontophoresis

**DOI:** 10.1016/j.ijpx.2025.100317

**Published:** 2025-02-10

**Authors:** Jianling Hu, Yue An, Weiqing Wang, Jing Yang, Wenxin Niu, Xiumei Jiang, Kun Li, Changzhao Jiang, Jincui Ye

**Affiliations:** a Key Laboratory of Neuropsychiatric Drug Research of Zhejiang Province, Institute of Materia Medica, Hangzhou Medical College, Hangzhou 310013, China; b Collaborative Innovation Center of Green Pharmaceuticals, Zhejiang University of Technology, Hangzhou 310014, China

**Keywords:** Transdermal drug delivery, Conductive hydrogel microneedles, Iontophoresis, Pioglitazone hydrochloride, Type II diabetes

## Abstract

The conventional oral administration of pioglitazone for Type II diabetes management is frequently compromised by hepatic first-pass metabolism and associated systemic adverse effects, necessitating the development of enhanced transdermal delivery approaches. This study developed a transdermal drug delivery system combining conductive hydrogel microneedles and iontophoresis to improve the transdermal delivery of pioglitazone hydrochloride (PIO) and its therapeutic efficacy in the treatment of type II diabetes. The microneedles, fabricated using poly(methyl vinyl ether-*alt*-maleic anhydride) as the main matrix material, exhibited excellent conductivity, mechanical strength, and high drug loading capacity. In vitro permeation experiments demonstrated that, when combined with iontophoresis at a current intensity of 0.5 mA, the cumulative permeation of PIO reached 238.1 ± 27.14 μg/cm^2^ within 48 h, significantly higher than that of the microneedle group alone. In a type II diabetic rat model, the microneedle-iontophoresis system displayed a significantly better hypoglycemic effect than the oral administration group, with a blood glucose reduction of 6.3 mmol/L on day 8, significantly higher than the 5.1 mmol/L reduction in the positive control group. Pharmacokinetic analysis indicated that the T_max_, T_1/2_, and mean residence time of the system were longer than those of oral administration, indicating sustained-release characteristics. Skin irritation tests revealed that the system caused only mild, transient skin irritation, with complete skin recovery within 24 h. In conclusion, conductive hydrogel microneedles combined with iontophoresis can effectively enhance PIO transdermal delivery, bioavailability, and therapeutic efficacy while also exhibiting good safety and potential clinical application value.

## Graphical abstract

**Figure:**
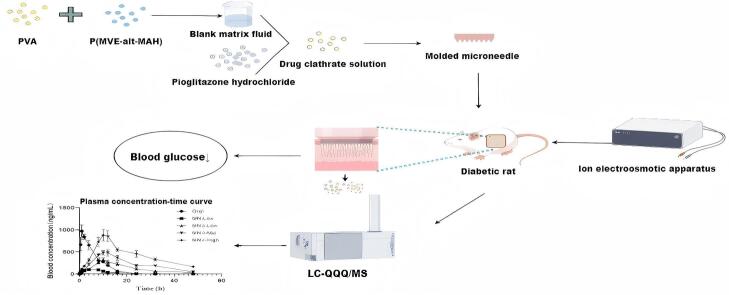

## 1 Introduction

Recently, transdermal drug delivery systems (TDDS) have garnered significant attention as a novel drug administration route(Richa et al., 2024). TDDS offers several unique advantages over traditional oral and injectable drug delivery, including the avoidance of hepatic first-pass metabolism, improved drug bioavailability, reduced gastrointestinal side effects and irritation, and the maintenance of stable plasma drug concentrations with reduced peak-valley fluctuations and associated toxicity. Moreover, TDDS are non-invasive and user-friendly, which increases patient compliance. These advantages make TDDS a promising alternative to conventional drug delivery methods(Rotake et al., 2024; Vaseem et al., 2024).

The stratum corneum, as the primary barrier to drug permeation in the skin, significantly limits the applications and efficacy of TDDS. The stratum corneum, which is composed of corneocytes and a lipid-rich matrix, has a compact structure that impedes the passive diffusion of most drugs(Matharoo et al., 2024). Even small, lipophilic molecules struggle to achieve high-dose transdermal delivery(Hassan et al., 2022). To overcome this barrier, researchers have developed several permeation enhancement techniques, including chemical enhancers, physical methods, and formulation strategies(Phaugat et al., 2023). While chemical enhancers can increase skin permeability by disrupting skin lipids or interacting with keratin, they may cause skin irritation or allergic reactions and are ineffective for macromolecular drugs(Hmingthansanga et al., 2022; Raghav et al., 2024). Therefore, It is critical to develop new methods that can facilitate the transdermal delivery of a wider range of drugs, particularly macromolecules and biologics.

Recently, physical permeation enhancement techniques, such as microneedles and iontophoresis, have gained considerable attention due to their ability to effectively improve transdermal drug absorption(Diwe et al., 2024; Umeyor et al., 2023). Compared with chemical enhancers, physical methods can significantly reduce drug absorption lag time, increase drug absorption, and improve permeation efficiency, thus broadening the range of drugs suitable for transdermal delivery(Sugumar et al., 2023).

Microneedle technology employs micron-scale needles that can painlessly penetrate the stratum corneum with minimal pressure, generating microchannels that bypass the skin barrier, allowing for effective delivery of small- and large-molecule drugs(Priya et al., 2023; Sultana et al., 2023). Many types of microneedles have been developed, including coated, solid, dissolving, hollow, and hydrogel microneedles(McNamee et al., 2023; Wang et al., 2023c). Hydrogel microneedles have demonstrated promising application prospects owing to their high drug-loading capacity, diversity in size and shape, and ability to be completely removed after use(Dhandapani et al., 2023; Liu et al., 2023a; Ashwini et al., 2023). Hydrogel microneedles have been widely explored in cancer treatment, wound healing, disease diagnosis, and vaccine delivery(Kanugo, 2023; Liu et al., 2023b; Wang et al., 2023a; Yang et al., 2024).

However, using microneedle technology alone may not achieve sufficient drug permeation depth and duration. To address this limitation, iontophoresis, an important physical permeation enhancement method, can be combined with microneedle technology. Iontophoresis uses a weak electric current to drive charged drug molecules through the skin and into local tissues or systemic circulation(Ramadan et al., 2023). Its main mechanisms include electromigration, electroosmosis, and electroporation(Andrade et al., 2023). This technique offers several advantages, including precise control of drug release rates, rapid onset of action, and the ability to overcome drug molecular weight limitations(De et al., 2021; Sugibayashi et al., 2021). Several iontophoresis-based transdermal drug delivery products have been approved and marketed, including LidoSite® for lidocaine and Ionsys® for fentanyl, confirming the clinical feasibility of this technology(Bakshi et al., 2020). However, high device costs, complex operation, and skin safety concerns hinder its widespread application(Fukuta et al., 2020; Nguyen and Vo, 2021). Integrating iontophoresis systems into wearable devices is a promising new direction in this field, enabling continuous and controlled drug delivery(Tan et al., 2022).

Combining microneedles with iontophoresis produces a significant synergistic effect on transdermal drug permeation(Junaid and Banga, 2022; Shivani and Babu, 2023; Shukla et al., 2022; Zheng et al., 2023). Microneedles disrupt the stratum corneum, reduce skin resistance, and promote initial drug permeation, while iontophoresis further drives drug molecules deeper into the skin and improves drug distribution. This combined approach holds great potential for delivering drugs that require higher doses or have larger molecular weights, potentially addressing limitations in current transdermal drug delivery.

Pioglitazone hydrochloride (PIO), an oral thiazolidinedione antidiabetic drug, reduces insulin resistance by activating peroxisome proliferator-activated receptor γ. It is widely used to treat type II diabetes. However, long-term oral pioglitazone administration may result in adverse reactions such as fluid retention, weight gain, and increased cardiovascular risk(Botrous et al., 2024). Transdermal administration can bypass hepatic first-pass metabolism, reduce peak blood drug concentrations, and decrease systemic exposure, potentially reducing these adverse reactions and improving treatment safety and patient compliance(Balakrishnan and Gopi, 2024). However, due to PIO's high hydrophilicity (logP = 2.3) and relatively large molecular weight (392.9 g/mol), traditional transdermal delivery methods, such as nanocarriers and chemical enhancers, face significant challenges in achieving effective drug permeation through the stratum corneum barrier(Obaidat et al., 2022; Ramkanth et al., 2021).Even with permeation enhancers like propylene glycol, the risk of skin irritation further limits the optimization of transdermal formulations(Nair et al., 2019). Consequently, developing new transdermal delivery strategies is crucial for the safe and effective application of pioglitazone.

Given this context, this study proposes a novel TDDS that enhances PIO transdermal delivery by fabricating conductive hydrogel microneedles and integrating them with iontophoresis. We investigated the formulation factors and iontophoresis parameters that influence drug permeation characteristics, aiming to elucidate the potential mechanisms of this combination approach and explore the synergistic effects of microneedles and iontophoresis in depth. Additionally, this study aimed to develop an efficient and safe transdermal delivery system for pioglitazone, offering a new strategy for the treatment of type II diabetes. Furthermore, this study provides a theoretical basis for the design of wearable electroporation devices and compatible drug formulations, potentially facilitating the development of efficient drug-device combination systems and advancing innovation in the field of TDDS.

## 2 Material and methods

### 2.1 Materials and instruments

PIO (purity ≥98 %, Yuanye Biotechnology Co., Ltd., Shanghai, China). Poly(methyl vinyl ether-*alt*-maleic anhydride) [P(MVE-alt-MAH), MW 80 kDa] and rhodamine B were obtained from Aladdin Co., Ltd. (Shanghai, China). Polyvinyl alcohol 124 (PVA 124) and PVA 350 were procured from Wokei Co., Ltd. (Shanghai, China). Polyvinylpyrrolidone (PVP, MW 1.3 MDa) and water-soluble chitosan (MW 30–60 k) were acquired from Macklin Co., Ltd. (Shanghai, China). Sodium chloride and ammonium acetate were supplied by Kelun Co., Ltd. (Chengdu, China). Sodium hyaluronate (HA, MW 1000 kDa) was obtained from Bloomage Biotechnology Co., Ltd. (Shandong, China), and hydroxypropyl methylcellulose (HPMC, MW 15 MDa) was acquired from Anhui Shanhe Pharmaceutical Excipients Co., Ltd. (Anhui, China). Rosiglitazone was sourced from TargetMoI Co., Ltd. (Shanghai, China). Methanol and acetonitrile were obtained from Tedia Company, Inc. (Ohio, USA). Purified water was acquired from Hangzhou Wahaha Group Co., Ltd. (Hangzhou, China).

High-Performance Liquid Chromatography (HPLC) (Agilent 1260,Agilent,America), HPLC-Tandem Mass Spectrometry (HPLC-MS/MS) (Agilent 6495C,Agilent, America), Fourier Transform Infrared (FTIR) Spectrometer (Nicolet iS 10,Thermofisher Scientific, America), Transmission Electron Microscope (TEM) (HT7700,HITACHI,Japan), Scanning Electron Microscope (SEM) (JSM-IT200, JEOL.,Japan), Texture Analyzer (TA.XT Plusl, Stable Micro Systems Texture Analyzer,Britain), Iontophoresis Drug Delivery Device (DS-UCMS1B,Ding Shi Medical,China), Ring Ammeter (VC140, VICTOR, China), Optical Microscope (DMi8, Leica, Germany), Freeze Dryer (LABCONCO Free Zone 6plus,Labconco, America), In Vivo Imaging System (IVIS) (France, Biospace, France), Glucometer (On Call EZ III, On Call, China).

### 2.2 Preparation and optimization of conductive hydrogel microneedles

#### 2.2.1 Fabrication of PIO conductive microneedles

To develop stable and conductive microneedle arrays with suitable mechanical strength, drug loading capacity, and biocompatibility for pioglitazone hydrochloride delivery. The microneedle arrays were fabricated using a polydimethylsiloxane (PDMS) mold, with the needle cavities designed as quadrangular pyramids. The microneedles were 850 μm long, 450 × 450 μm in base area, and 900 μm apart. The microneedles were arranged in a 15 × 15 array (Demir et al., 2017).

The PIO-loaded hydrogel matrix solution was prepared as follows: Microneedle matrix materials were continuously stirred in purified water at 90 °C until fully dissolved, yielding a homogeneous, moderately viscous, pale yellow solution. After cooling the solution to 25 ± 2 °C, a specified amount of PIO was added. The mixture was agitated at 500 rpm for 2 h at 25 °C to ensure complete dissolution of the drug, yielding the drug-loaded hydrogel matrix solution.

The PIO conductive hydrogel microneedles were fabricated using a casting method. The PIO-loaded gel matrix solution was poured into the PDMS molds. To ensure complete filling of the microneedle cavities, the molds were degassed at −0.1 MPa and 25 °C for 20 s using a vacuum pump. Excess solution was scraped from the mold surface with a spatula. To facilitate initial gelation, the molds were dried at room temperature for 10 h. Subsequently, the molds were oven-cured at 50 °C for 3 h. After curing, the microneedle arrays were carefully demolded, yielding the PIO-loaded conductive hydrogel microneedles.

#### 2.2.2 Microneedle matrix material selection and optimization

To develop conductive hydrogel microneedles with improved conductivity and high drug loading capacity, several matrix materials, including PVP, HA, water-soluble chitosan, HPMC, and P(MVE-alt-MAH), were evaluated. To prepare the matrix solutions, each polymer was dissolved in purified water at a concentration of 5 % (*w*/*v*) and stirred at 500 rpm at 25 °C until complete dissolution.

The electrical conductivity of the matrix materials was assessed by immersing the matrix solutions in the positive and negative poles of an iontophoresis drug delivery device and measuring the current intensity with a ring ammeter under different matrices. Moreover, to evaluate the solubility of PIO in each matrix, an excess amount of drug was added to the matrix solutions and stirred at 500 rpm for 24 h at 25 °C. Subsequently, the solutions were filtered through a 0.45 μm membrane, and the drug concentration in the filtrate was determined using high-performance liquid chromatography (HPLC).

The HPLC conditions were as follows: Agilent ZORBAX Eclipse XDB-C18 column (4.6 × 150 mm, 5 μm); mobile phase of 0.05 mol/L ammonium acetate buffer and methanol (35:65, *v*/v); flow rate of 1.0 mL/min; column temperature of 30 °C; detection wavelength of 266 nm; injection volume of 10 μL(Marie et al., 2023).

To improve the mechanical properties of the microneedles, HA, PVP, and PVA were selected as toughening agents and blended with P(MVE-alt-MAH) at weight ratios of 1:15, 1:10, and 1:5, respectively and their respective weight ratios are detailed in **Table S1**, to prepare composite matrices. Each toughening agent was dissolved in purified water and mixed at 500 rpm at room temperature until complete dissolution. Subsequently, P(MVE-alt-MAH) was added, and the mixture was heated to 90 °C and stirred for 2 h to obtain a homogeneous solution. After cooling to room temperature, 1 % (*w*/*v*) PIO was added, and the solution was stirred for an additional 1 h to ensure complete dissolution of the drug.The mechanical strength of the microneedles was the primary consideration when optimizing the matrix materials. The Parafilm M® test served as a rapid, qualitative screening tool to verify penetration capability. Subsequently, a texture analyzer provided precise, quantitative mechanical strength measurements to confirm the chosen formulation's mechanical integrity. The initial mechanical performance evaluation was conducted using a Parafilm M® puncture test, in which the microneedle array was manually pressed into an 8-layer stack of Parafilm M® with a constant force of approximately 5 N, calibrated using an analog push-pull gauge (NK-50), for 30 s(Sulistiawati et al., 2024). The number of layers penetrated was recorded. Formulations that penetrated fewer than three layers were considered mechanically insufficient and eliminated. Further mechanical testing was performed using a texture analyzer equipped with a 25-mm diameter flat-head cylindrical probe. Microneedle patches were cut into 3 × 3 microneedle units, with the test parameters set as follows: pre-test speed of 1.0 mm/s, test speed of 0.5 mm/s, post-test speed of 1.0 mm/s, trigger force of 0.049 N, and compression distance of 1.0 mm. During testing, the probe compressed the microneedles at a constant speed to the set distance, and the force-displacement curve was recorded. The maximum compression force before microneedle deformation or breakage was calculated using the Texture Exponent software.

Matrix material combinations exhibiting optimal mechanical strength, toughness, conductivity, and drug loading capacity were selected to ensure the structural integrity and effective skin penetration of the microneedles.

### 2.3 Characterization of PIO conductive microneedles

#### 2.3.1 Drug solubility characteristics in the matrix solution

Given the poor water solubility of PIO, this study aimed to investigate its physicochemical interactions with the microneedle carrier P(MVE-alt-MAH) to elucidate the drug loading mechanism. Fourier transform infrared (FTIR) spectroscopy was used to analyze potential hydrogen bonding, crosslinking, or encapsulation phenomena between the drug and the carrier.

P(MVE-alt-MAH) and PIO were dissolved in purified water separately and mixed to form a homogeneous solution. An appropriate amount of the solution was rapidly frozen at −80 °C and lyophilized using a freeze-dryer to produce a dry powder sample. The lyophilized sample was ground into a fine powder and mixed with spectroscopic-grade KBr at a 1:100 ratio to form a pellet. FTIR spectra of the samples were recorded in the range of 400–4000 cm–1 using an FTIR spectrometer. The control groups, including pure P(MVE-alt-MAH), pure PIO, and a 1:1 (*w*/w) physical mixture, were prepared under identical conditions to ensure comparability.

Transmission electron microscopy (TEM) was used to analyze the dispersion state of PIO in the matrix. To prepare a stock solution, approximately 10 mg of the lyophilized sample was dissolved in 1 mL of deionized water and stirred at 500 rpm for 30 min at 25 °C. The solution was diluted 20-fold and drop-cast onto a carbon film-supported copper grid. After 2 min of adsorption, excess liquid was removed with filter paper, and the grid was air-dried at room temperature. TEM imaging was performed at an accelerating voltage of 90 kV to observe the morphology and distribution of the drug in the matrix.

#### 2.3.2 Morphological characterization

A multi-level imaging approach was used to evaluate the morphological characteristics of the microneedles. At the macroscopic level, images of the microneedle array were captured using a digital camera, and the overall size, shape, and arrangement consistency were observed. At the microscopic level, an optical microscope was used to examine the microneedle details, focusing on the sharpness of the needle tips and the smoothness of the needle bodies. Furthermore, scanning electron microscopy (SEM) was used to perform high-precision scanning analyses of the microneedle surface texture, tip sharpness, and microstructure.

The SEM was employed to characterize the surface and internal morphology of the MNs. Prior to imaging, the SEM instrument (JSM-IT200, JEOL, Japan) was preheated for 30 min to ensure stable operating conditions. A small quantity of the fabricated MNs was then affixed to a conductive carbon tape on an SEM sample stub. To enhance conductivity and prevent charging artifacts during imaging, the samples were sputter-coated with a thin layer of gold using a small ion sputtering instrument. The gold-coated MNs were subsequently transferred to the preheated SEM chamber. Images were acquired at an accelerating voltage of 15.0 kV and a working distance of 10.4 mm. These parameters were maintained throughout the imaging process to ensure consistency when observing both the surface and internal morphology of the MN samples.

#### 2.3.3 Skin penetration performance

Aimed to evaluate the ability of the microneedles to penetrate rat skin and create drug delivery pathways while minimizing damage. The skin penetration ability of the microneedles was assessed in vitro using rat skin. Frozen skin samples were thawed and rinsed with physiological saline, and surface moisture was removed before fixing the skin to a polystyrene foam board. The microneedle patch was vertically pressed onto the skin with a force of 5 N for 30 s and then removed.

To fix the treated skin samples, they were immersed in a 4 % paraformaldehyde solution for 24 h, embedded in paraffin, and sectioned. The sections were stained with hematoxylin and eosin (H&E) and observed under a digital microscope to evaluate the microneedle penetration effect. To determine whether the microneedles penetrated the stratum corneum into the dermis, the skin was stained with a 0.4 % trypan blue solution for 5 min and rinsed with physiological saline after repeated puncture experiments. The skin penetration ability of the microneedles and their successful formation of channels reaching the effective drug delivery depth were visually assessed based on the staining results.

#### 2.3.4 Swelling performance

To evaluate the swelling behavior and morphological changes of hydrogel microneedles in vivo and in vitro. Hydrogel microneedles swell when exposed to water. We evaluated the swelling behavior of the microneedles under in vivo and in vitro conditions. The microneedle array was cut into fragments containing 7 × 7 needle tips and pressed onto the shaved back skin of rats with a force of 5 N for 30 s, followed by fixation with 3 M tape. The microneedles were removed at predetermined intervals of 0, 5, 10, 30, 45, and 60 min, and their morphological changes and swelling were observed using a microscope.

For in vitro swelling evaluation, the microneedle tips were immersed in phosphate-buffered saline (PBS, pH 7.4) at room temperature and removed at 0, 10, 20, 30, 45, and 60 s. The surface liquid was removed with filter paper, and the swelling degree was measured and recorded under the same microscopic conditions as in the in vivo study. The change in microneedle length was measured using ImageJ software, and the swelling ratio was calculated using the formula:

$$\text{Swelling Ratio}=\left(\frac{L_{t}-L_{0}}{L_{0}}\right)\times 100\%$$

Where L_t_ is the microneedle length after swelling and L_0_ is the initial length.

#### 2.3.5 Conductivity of PIO microneedles

Evaluation of conductivity properties of conductive microneedles and their precursor solutions.The conductivity of the conductive microneedles and their precursor solutions was assessed using an iontophoresis drug delivery device (Nanjing Dingshi Medical Equipment Co., Ltd.). Specifically, 500 mg of blank microneedle solution was poured into a pre-designed mold, and the positive and negative electrodes of the iontophoresis device were inserted to establish a conductive pathway. The current flowing through the solution was measured using a ring ammeter as a conductivity reference. The blank microneedles were immersed in deionized water for 60 s to allow swelling, removed, and fixed onto a backing substrate. The electrodes were connected to the swollen microneedle array under the same voltage and measurement conditions, and the current passing through the microneedles was recorded. To ensure that the results were comparable, the contact method and electrode parameters were kept consistent throughout the measurement process.

#### 2.3.6 Water content analysis

The water content of drug-loaded microneedles was measured to evaluate its water stability.Three batches of drug-loaded microneedles were prepared, and three samples from each batch were selected to evaluate moisture content. The initial mass (m_0_) of each sample was accurately weighed and recorded. Microneedle samples were dried under the same vacuum and temperature conditions as used in the manufacturing process (60 °C for 24 h) to ensure consistency between manufacturing and testing conditions.After drying, the samples were cooled to room temperature in a desiccator for 30 min, and their mass (m_1_) was weighed. The drying and weighing process was repeated until the difference between two consecutive weighings was less than 0.5 mg, yielding the constant mass (m_f_). The moisture content was calculated using the formula:

$$\text{Water Content}=\left(\frac{m_{0}-m_{f}}{m_{0}}\right)\times 100\%$$

#### 2.3.7 Drug distribution uniformity

To evaluate the uniformity of drug distribution within the microneedles, a solution containing 1 % (*w*/w) PIO was prepared as the model drug. A homogeneous 10 g solution was formulated by dissolving 100 mg of PIO in 9900 mg of blank microneedle solution. Complete dissolution was ensured by stirring the mixture at 500 rpm for 2 h at 25 °C. Microneedle patches were then fabricated for drug distribution uniformity testing by transferring 500 mg aliquots of the 1 % PIO-loaded solution (containing 5 mg of PIO each) into PDMS molds.

The microneedle array was carefully sectioned into designated regions using a sterile surgical blade under magnification (10×). This procedure was performed on a clean, flat surface with careful attention to maintaining the structural integrity of individual microneedles during the cutting process. Each part was immersed in 80 % methanol and extracted by ultrasonication for 1 h. After filtration, the drug concentration was analyzed using HPLC. The microneedle patch was divided into nine regions (four corners and five centers), and drug content was extracted and analyzed separately for each region. The average value and standard deviation of the drug content across regions were calculated. The coefficient of variation (CV%) was used to assess drug distribution uniformity.

### 2.4 Investigation of influencing factors in the microneedle-iontophoresis drug delivery system

#### 2.4.1 In vitro skin permeation test

To investigate the transdermal delivery efficiency of pioglitazone hydrochloride using the microneedle-iontophoresis system compared to microneedles alone. In vitro permeation experiments were conducted using Franz diffusion cells to simulate the PIO transdermal drug delivery process. Each Franz diffusion cell had an effective diffusion area of 0.77 cm^2^, and skin samples were obtained from male Sprague-Dawley (SD) rats (200–250 g). After the rats were euthanized, full-thickness skin from the dorsal region was immediately excised, excess fat and connective tissue were removed, and the skin was rapidly frozen at −80 °C for later use. Before use, the frozen skin samples were thawed in physiological saline at 37 °C for 3–5 min to restore their physiological state.

MNs for in vitro skin permeation studies were cut into fragments containing 7 × 7 microneedles. The total drug loading was approximately 1.60 mg, with about 0.15 mg in the microneedle shafts and 1.45 mg in the base layer. The microneedle array was vertically inserted into the excised skin with a force of 10 N and pressed for 1 min to ensure sufficient penetration and avoid excessive damage.

**Fig. 1:**
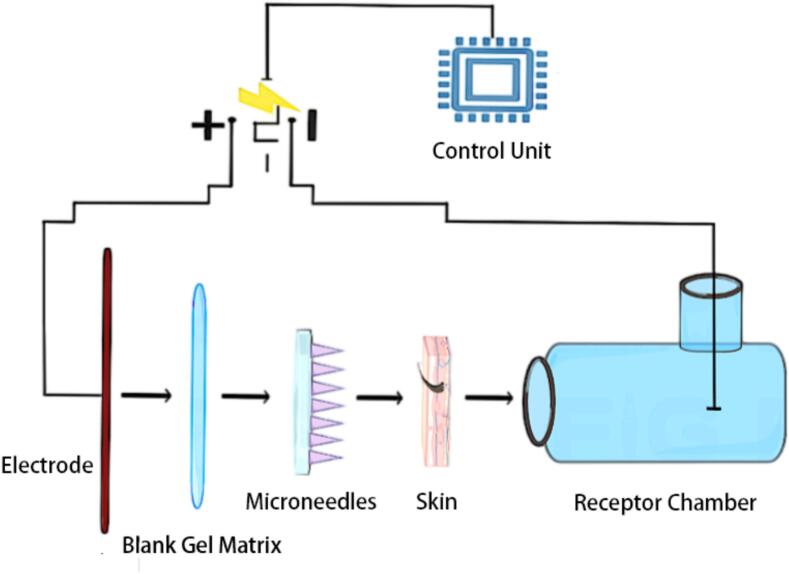
Schematic diagram of the microneedle-iontophoresis drug delivery system.

To enhance conductivity, a blank hydrogel matrix comprising 2.5 % (*w*/w) P(MVE-alt-MAH) and 97.5 % (w/w) purified water was coated on the positive electrode of the iontophoresis device to ensure complete contact between the electrode and the base of the microneedles. To complete the circuit connection, a platinum electrode was used as the negative electrode and immersed in the receptor solution in the receptor compartment **(**Fig. 1**)**. To maintain drug solubility, 5 mL of 20 % (*v*/v) PEG 400 and 0.9 % NaCl was added to the receptor compartment as the receptor solution. Before starting the experiment, no air bubbles were allowed to form between the skin and the receptor solution to avoid altering the permeation results. To simulate normal human skin temperature, the receptor compartment was maintained at 32.0 ± 0.5 °C using a constant-temperature circulating water bath.

During the experiment, six sampling points were established at 1, 2, 4, 6, 8 and 10 h to ensure the attainment of steady-state conditions and the linearity of the permeation curve. At each sampling interval, 1 mL samples were withdrawn from the receptor compartment and replaced with an equal volume of pre-warmed fresh receptor solution. The collected samples were filtered through a 0.22 μm membrane filter, and the amount of PIO that permeated the skin was quantified using HPLC. The cumulative permeation amount (Q) was calculated using the formula:

$$Q=\frac{1}{S}\left[C_{n}\times V+\sum_{i=1}^{n-1}C_{i}\times V_{i}\right]$$

where C_n_ is the drug concentration in the receptor compartment at the n^th^ sampling time, V is the total receptor volume, C_i_ is the concentration at each previous sampling time, V_i_ is the volume of sampled receptor fluid each time, and S is the effective diffusion area.

#### 2.4.2 Influencing factors of iontophoresis parameters

The effectiveness of iontophoresis is mainly influenced by parameters such as current intensity, NaCl concentration, matrix pH value, and drug concentration. This study systematically investigated the factors influencing the conductive microneedle-iontophoresis system.

Effect of current intensity: In vitro skin permeation experiments with Franz diffusion cells were performed using PIO-loaded conductive hydrogel microneedles. Current intensities of 0.1, 0.3, and 0.5 mA were set based on the human tolerance range and the principle of balancing iontophoresis efficiency reported in the literature (Abe et al., 2023), and the current was applied continuously for 10 h (*n* = 3). Receptor solution samples were collected and drug concentrations were determined using HPLC to evaluate the effect of different current intensities on cumulative permeation.

Effect of NaCl concentration: NaCl was added into the microneedle formulation at concentrations of 0 %, 0.5 %, and 1.0 % (*w*/w) to improve microneedle conductivity and iontophoresis efficiency. PIO microneedles were prepared using conductive hydrogel matrices with different NaCl concentrations, and in vitro skin permeation experiments were conducted at a constant current intensity of 0.5 mA (n = 3). Cumulative drug permeation was evaluated to determine the optimal NaCl concentration.

Effect of pH value: The pH value of the microneedle precursor solution was adjusted to 5.0, 6.0, and 7.0 using 1 M NaOH, considering drug and microneedle material stability. The appearance, mechanical properties, and stability of the microneedles were evaluated at different pH values, and the effect of pH on permeation efficiency was assessed to ensure suitability for iontophoretic drug delivery.

Effect of drug concentration: Microneedle formulations with PIO concentrations of 0.5 %, 1.0 %, and 2.0 % (w/w) were prepared, ensuring that the drug did not precipitate in the matrix and maintaining formulation stability and microneedle moldability. In vitro skin permeation experiments were conducted at a current intensity of 0.5 mA (n = 3 per group). Cumulative permeation was determined using HPLC, and the relationship between drug concentration and permeation efficiency was analyzed.

After optimizing the aforementioned parameters, the optimized PIO microneedles were selected for further in vitro skin permeation experiments. During the 48-h experiment, receptor solution samples were collected at 2, 4, 6, 8, 12, 24, 32, and 48 h. The cumulative permeation curves of the iontophoresis and control groups without current application (0 mA) were compared to evaluate the enhancing effect of iontophoresis on transdermal drug delivery.

### 2.5 In vivo diffusion characteristics of the microneedle-iontophoresis drug delivery system

To simulate the in vivo diffusion behavior of PIO in the skin, rhodamine B was used as a substitute marker. Rhodamine B is a cationic fluorescent dye with a molecular weight comparable to that of PIO, making it a suitable drug diffusion simulant. To prepare the fluorescent microneedles, 2.0 g of blank microneedle precursor solution was mixed with 100 μL of rhodamine B solution at a concentration of 2 mg/mL and thoroughly stirred. The mixture was centrifuged at 6000 rpm for 10 min to remove air bubbles and ensure uniform distribution of rhodamine B. Subsequently, the mixed solution was injected into the microneedle mold to fabricate the fluorescent microneedle array.

For the in vivo diffusion experiment, six male SD rats (200 ± 50 g) were randomly divided into the iontophoresis group (*n* = 3) and the control group (n = 3). Before the experiment, the hair on the back and sides of the rats was removed using an electric hair clipper. Subsequently, the fluorescent microneedle patch was vertically pressed onto the dorsal skin of the rats with a force of 10 N for 1 min to ensure sufficient insertion.

The base of the microneedle patch in the iontophoresis group was connected to the iontophoresis device, and a constant current of 0.5 mA was applied for drug delivery. The control group was connected to the same device; however, the current was set to 0 mA. At 3, 30, and 60 min after drug administration, the rats were placed in an in vivo imaging system (IVIS) for fluorescence imaging using an excitation wavelength of 540 nm and an emission wavelength of 620 nm. The fluorescence intensity and diffusion area were quantitatively analyzed from the fluorescence images to evaluate the enhancing effect of iontophoresis on rhodamine B transdermal diffusion.

### 2.6 Pharmacodynamics of microneedle-iontophoresis drug delivery system

#### 2.6.1 Construction of a type II diabetic rat model

To evaluate the hypoglycemic efficacy of PIO microneedles combined with iontophoresis, a type II diabetic rat model was established using a high-fat diet and low-dose streptozotocin (STZ)(Annaas Budi, 2024). Male SD rats weighing 200 ± 50 g and aged 6–8 weeks were selected for the experiment.

The rats were given a high-fat diet for 4 weeks. Subsequently, STZ was dissolved in 0.1 M sodium citrate buffer (pH 4.5) in an ice bath to prepare a fresh 1 % STZ solution, which was used within 30 min. The model group rats received a single intraperitoneal injection of STZ at a dose of 35 mg/kg(Wang et al., 2016).

These rats were kept under standard experimental conditions for 2 weeks, with free access to food and water. Fasting blood glucose levels were measured using a glucometer. Rats with fasting blood glucose levels exceeding 12 mmol/L and diabetic symptoms such as polydipsia, polyphagia, and polyuria were confirmed as successful type II diabetic models.

#### 2.6.2 Pharmacodynamic experiments

To evaluate the hypoglycemic efficacy of the microneedle-iontophoresis system in a type II diabetic rat model and compare it to conventional oral pioglitazone administration. The hypoglycemic effect of the PIO microneedle-iontophoresis system was assessed by comparing changes in fasting blood glucose levels before and after administration**(Fig. S1)**. Before administration, fasting blood glucose levels were recorded after a 12-h fast. Subsequently, the corresponding treatments were administered, and fasting blood glucose was measured at 0, 2, 4, 6, 8, 12, and 24 h post-administration to plot blood glucose fluctuation curves. The blood glucose change (Δ Blood Glucose, mmol/L) was defined as the difference between fasting blood glucose levels at 6 h pre- and post-administration, calculated as follows:

$$∆\text{Blood Glucose}\left(\text{mmol}/L\right)=A-B$$

where A is the fasting blood glucose level pre-administration, and B is the fasting blood glucose level post-administration. ∆ > 0 indicates a significant hypoglycemic effect, while ∆ ≤ 0 indicates no effect.

Thirty rats were randomly divided into four groups (*n* = 6). Model: diabetic rats that did not receive any treatment and were used to evaluate the natural progression of hyperglycemia; oral administration: diabetic rats administered a dose of 1.58 mg/kg of commercially available PIO tablets by gavage to ensure a significant hypoglycemic effect; iontophoresis microneedle: diabetic rats treated with PIO microneedles combined with iontophoresis. The microneedle patch was applied to the shaved dorsal region of the rats with a force of approximately 10 N for 1 min to ensure sufficient insertion. A hydrogel matrix was placed on the back of the microneedles, and the positive electrode pad was placed on the microneedle patch while the negative electrode pad was fixed on the abdominal region. The transdermal drug delivery device was connected, and a constant current of 0.5 mA was supplied for 6 h. The PIO transdermal dose was set at 10 mg/kg based on the 14 % drug content in the microneedle tips relative to the total dose, and the conventional microneedle group received the same microneedle application treatment as the experimental group but without the current application. The current was set to 0 mA and the dose at 10 mg/kg.

Throughout the experiment, the body weight, food and water intake, and behavioral performance of the rats were recorded. Blood glucose measurement results are expressed as mean ± standard deviation. Statistical analysis was performed using Statistical Package for the Social Sciences software (version 25.0). Normality and homogeneity of variance tests were conducted. If the conditions were met, analysis of variance (ANOVA) and Tukey's multiple comparison tests were used. A *P* < 0.05 was considered statistically significant.

#### 2.6.3 Skin irritation test

To evaluate the potential skin irritation of the conductive hydrogel microneedle-iontophoresis system, male SD rats weighing 200–250 g were used as the animal model. The hair on the dorsal region of each rat was removed 24 h before the experiment using an electric hair clipper.

The microneedle patch was applied to the shaved area with a force of approximately 10 N for 1 min to ensure sufficient microneedle penetration into the skin. A hydrogel matrix was placed on the back of the microneedle patch to promote microneedle swelling and enhance contact with the skin. The electrode pads were placed on the hydrogel layer and connected to the iontophoresis drug delivery device, with the current intensity set at 0.5 mA. The microneedle-iontophoresis system was kept in place for 8 h. After the treatment, the microneedle patch and electrodes were carefully removed.

Before patch removal (baseline) and at 0, 6, 24, and 48 h after removal, the application site was observed and assessed for signs of skin irritation, including erythema (redness), edema (swelling), and erosion. The Draize scoring system was used to evaluate the degree of skin irritation, with the severity of erythema and edema scored from 0 (no reaction) to 4 (severe reaction).

To assess cumulative skin irritation and potential histopathological changes, the respective treatments were administered daily for 7 consecutive days following the same protocol. After the treatment period, skin tissue samples from the application site were collected for histological examination, and the average dorsal skin thickness of male SD rats is approximately 1.35–1.42 mm. The tissues were fixed in 10 % neutral buffered formalin, dehydrated, embedded in paraffin, and sectioned into 5 μm slices. H&E staining was performed, and the degree of inflammatory cell infiltration was observed under an optical microscope.

### 2.7 Pharmacokinetics of microneedle-iontophoresis drug delivery system

#### 2.7.1 Determination of plasma drug concentrations

In this study, the concentration of PIO in rat plasma was determined using an HPLC-tandem mass spectrometry (MS/MS) method, with rosiglitazone as the internal standard. The operating procedures were as follows: A 50-μL aliquot of rat plasma sample was spiked with 10 μL of rosiglitazone internal standard solution at a concentration of 100 ng/mL. Subsequently, 200 μL of acetonitrile was added, and the mixture was thoroughly vortexed for 1 min to precipitate proteins. The sample was centrifuged at 12,000 rpm for 10 min, and the supernatant was filtered through a 0.22-μm microporous membrane and transferred to a sample vial for HPLC-MS/MS analysis.

The chromatographic and mass spectrometric conditions were as follows:

Chromatographic conditions: Column: Poroshell 120 EC-C18 (4.6 × 100 mm, 2.7 μm); Mobile phase: gradient elution using 0.1 % formic acid aqueous solution (A) and acetonitrile (B) as presented in **Table S2**; flow rate: 0.6 mL/min; column temperature: 40 °C; injection volume: 10 μL; and autosampler temperature: 4 °C.

Mass spectrometric conditions: ESI in MRM mode; Ion spray voltage: 4000 V; Ion source temperature: 350 °C; PIO: *m*/*z* 357.1 to 134.1 (collision energy: 33 eV); and Rosiglitazone: m/z 358.0 to 135.1 (collision energy: 30 eV).

#### 2.7.2 Pharmacokinetic evaluation of conductive hydrogel microneedles at different dosages

To eliminate the impact of gender differences on the results, male SD rats weighing 200 ± 20 g were used to evaluate the pharmacokinetic characteristics of PIO administered via the conductive hydrogel microneedle-iontophoresis system. The rats were divided into six groups: high-dose microneedle-iontophoresis (MN-I-High, 30 mg/kg); medium-dose microneedle-iontophoresis (MN-I-Mid, 20 mg/kg); low-dose microneedle-iontophoresis (MN-I-Low, 10 mg/kg); microneedle control (MN-Low, 10 mg/kg); and oral (1.58 mg/kg).

To ensure proper insertion of the microneedles into the skin, the microneedle patch containing the specified dose was applied to the shaved dorsal region of the rats with approximately 10 N force for 1 min. A blank hydrogel matrix layer was placed on the back of the microneedle patch, with the positive electrode pad fixed on top of it and the negative electrode pad placed on the contralateral abdominal region. The electrodes were secured using medical adhesive tape. For the iontophoresis groups, an iontophoresis device was used to deliver a constant current of 0.5 mA for 8 h. The control and blank control groups had the current set to 0 mA. The positive control group received PIO suspension (1.58 mg/kg) by gavage in a 0.5 % carboxymethylcellulose sodium solution.

Blood samples were collected from the tail vein of each group at 0.5, 1, 2, 4, 6, 8 (withdrawal), 10, 12, 16, 24, 32, and 48 h post-administration. The tail was pre-warmed before blood collection, and blood samples were collected using micro-blood collection tubes and deposited in heparinized micro-centrifuge tubes. The samples were immediately centrifuged at 4000 rpm for 10 min to separate the plasma. Plasma samples were stored at −80 °C, avoiding repeated freeze-thaw cycles, until analysis.

For each plasma sample, 90 μL of rat blood and 10 μL of an internal standard (IS) solution (15 μg/mL rosiglitazone) were combined with 200 μL of a protein-precipitation solution (e.g., acetonitrile). The mixture was vortexed for 2 min, then centrifuged at 12,000 rpm for 10 min at 4 °C. A 100 μL aliquot of the supernatant was transferred for HPLC-MS/MS analysis. And the HPLC-MS/MS method was validated according to FDA guidelines, confirming specificity, linearity, accuracy, precision and LLOQ.

The validated HPLC-MS/MS method was used to determine PIO concentration in plasma. Key pharmacokinetic parameters, including AUC, C_max_, T_max_, T_1/2_, and mean residence time (MRT_0–∞_), were calculated using Phoenix WinNonlin software (version 8.3) via non-compartmental model analysis.

## 3 Results and discussion

### 3.1 Preparation and optimization of PIO conductive microneedles

To evaluate the conductivity of 5 % (*w*/w) PVP, HA, water-soluble chitosan, HPMC, and P(MVE-alt-MAH) matrix solutions, the current intensity was measured at the same output power. The results are presented in Table 1. The findings demonstrated that P(MVE-*alt*-MAH) exhibited significantly higher conductivity than the other polymers, mainly attributed to its unique alternating copolymer structure and highly reactive maleic anhydride groups. Upon hydrolysis, this structure generates numerous ionizable carboxyl groups, forming high-density charge carriers(Türk et al., 2010), thereby enhancing conductivity through ion migration and proton hopping mechanisms(Sun et al., 2022; Yang et al., 2021).

**Table 1:** Conductivity performance of different microneedle matrix materials.

Matrix Solution	Maximum Current (mA)
Water-soluble chitosan	1.13 ± 0.14
PVP	0.02 ± 0.01
HA	0.03 ± 0.01
P(MVE-alt-MAH)	2.99 ± 0.13
HPMC	1.21 ± 0.11

When assessing the drug loading capacity of the matrix solutions, we discovered that PIO solubility in water and alcoholic solutions was low, making it difficult to effectively dissolve in PVP, HA, water-soluble chitosan, and HPMC. Conversely, PIO exhibited substantial solubility in the P(MVE-alt-MAH) matrix solution. For further comparison, the solubility of the drug in purified water, anhydrous ethanol, PBS (pH = 7.4), and physiological saline was determined. The results are displayed in **Table S3**. Findings indicated that PIO solubility in the P(MVE-alt-MAH) solution was significantly higher than in the other solutions, suggesting that this polymer exerts a strong solubilizing effect on the drug. This may be attributed to hydrogen bonding or other non-covalent interactions between P(MVE-alt-MAH) and PIO or the formation of self-assembled structures, such as micelles, encapsulating the drug molecules and thereby enhancing its apparent solubility(Ma et al., 2016).

Based on conductivity performance and drug loading capacity, P(MVE-alt-MAH) was selected as the primary matrix material for PIO microneedles. However, microneedles made only from P(MVE-alt-MAH) were prone to fragility during the demolding process. To improve mechanical strength, PVP, PVA, and HA were screened as auxiliary matrix materials. The results revealed that drug precipitation occurred when PVP was mixed, while HA exhibited brittleness issues.

This study investigated the influence of varying PVA auxiliary matrix ratios on the penetration performance and mechanical strength of [P(MVE-alt-MAH)] microneedles. Results(**Table S4**) indicated that both PVA 124 and PVA 350 exhibited limited penetration capability and insufficient mechanical strength at lower ratios. Increased ratios enhanced penetration for both PVA types; however, PVA 350 demonstrated significantly superior mechanical strength, peaking at 39 N at a 10:1 ratio, which facilitated effective penetration of 3 Parafilm layers. Notably, while maintaining penetration depth at a 15:1 ratio, the mechanical strength of PVA 350 decreased considerably, suggesting that excessive auxiliary matrix can compromise the structural integrity of the microneedles. Consequently, the 10:1 ratio of P(MVE-alt-MAH) with PVA 350 offers an optimal balance between penetration capability and mechanical robustness,achieving a load-bearing pressure of 39 N (3 × 3 microneedle array), representing an ideal formulation for fabricating high-performance microneedles.

Based on the experimental results of conductivity, drug solubility, and mechanical strength, P(MVE-alt-MAH) emerged as the primary matrix material for PIO microneedles. The formulation combining P(MVE-alt-MAH) and PVA 350 at a 10:1 ratio demonstrated the best mechanical performance and penetration ability, significantly outperforming formulations that included PVA 124 and other toughening agents.

### 3.2 Characterization of PIO conductive microneedles

#### 3.2.1 Dissolution characteristics of PIO in P(MVE-alt-MAH)

To evaluate the solubility and distribution characteristics of PIO in the P(MVE-alt-MAH) microneedle system, various characterization techniques were employed to elucidate the interactions between the drug and the polymer matrix and their impact on drug solubility and release properties.

**Fig. 2:**
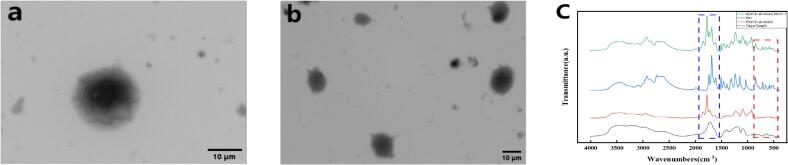
Characterization of PIO distribution and molecular interactions: (a-b) TEM images showing uniform dispersion; (c) FTIR spectra indicating hydrogen bonding.

TEM images **(**Figs. 2a-b**)** revealed that PIO was uniformly dispersed in the microneedle matrix as near-spherical particles, primarily within the diameter range of 10 to 20 μm. This uniform dispersion is favorable for achieving consistent drug release, improving stability, reducing burst release effects, and maintaining long-term therapeutic concentrations.

Fig. 2c shows the FTIR spectra used to investigate the molecular interactions between PIO and P(MVE-alt-MAH). In the 1500–2000 cm^−1^ range, the P(MVE-alt-MAH) carbonyl stretching and possible anhydride-related absorption bands were notably changed in the encapsulated sample, suggesting that the maleic anhydride rings may have partially opened or formed hydrogen bonds with PIO. The characteristic aromatic out-of-plane bending and ring-breathing peaks of PIO in the 500–800 cm^−1^ range disappeared, implying that the local environment of PIO's aromatic rings changed upon complexation due to its restricted vibrations. These alterations confirm that strong hydrogen bonding or other non-covalent interactions take place between PIO and P(MVE-alt-MAH), forming stable complexes. As a result, these complexes can reduce burst drug release, enhance formulation stability, control release kinetics, and ultimately improve PIO's solubility and bioavailability.

#### 3.2.2 Morphological characterization of PIO conductive microneedles

To further evaluate the structural integrity and morphological characteristics of the microneedle arrays, multi-scale characterization was performed using a digital camera, optical microscope, and SEM.

**Fig. 3:**
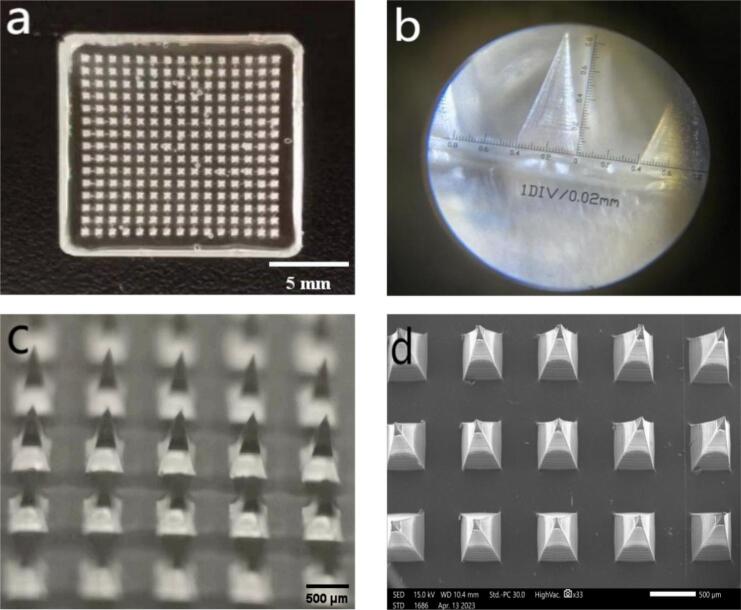
Microneedle appearance characteristics: a. Digital camera image; b. Image captured by optical magnifier; c. Microscope image at 16× magnification; d. SEM.

Digital camera images **(**Fig. 3a**)** revealed that the microneedle arrays were neatly arranged with uniform needle structures and a smooth backing layer, exhibiting no evident defects, which indicates a stable and controlled fabrication process. High-magnification images **(**Fig. 3b**)** demonstrated the sharpness of individual needle tips and their smooth surface, confirming the absence of cracks or damage. Optical microscope measurements **(**Fig. 3c**)** revealed that the microneedles exhibited a diameter of approximately 0.42 mm, a length of approximately 0.85 mm, and a tip angle of approximately 14.5°. Compared to other hydrogel-based microneedles(Turner et al., 2021), the PIO conductive microneedles exhibit a similar tip sharpness but offer a slightly taller needle length, which may facilitate deeper insertion and minimize the required insertion force. Indicating good penetration properties. SEM images **(**Fig. 3d**)** validated the quadrangular pyramidal structure and smooth surface of the microneedles, which enhances the uniform distribution of the drug within the skin.

**Fig. 4:**
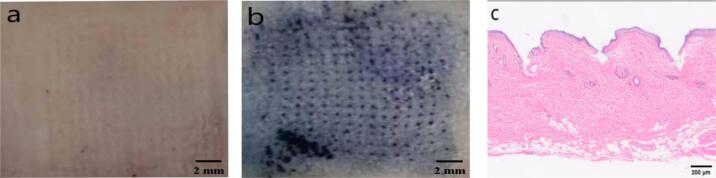
Skin penetration ability of microneedles: a. Before trypan blue staining; b. After trypan blue staining; c. Microneedle-pierced skin section. (For interpretation of the references to colour in this figure legend, the reader is referred to the web version of this article.)

H&E and trypan blue staining were used to evaluate the skin penetration performance of the microneedles. H&*E*-stained sections **(**Fig. 4c**)** indicated successful penetration through the stratum corneum, with the microneedles reaching the expected depth while maintaining intact surrounding tissue structures and minimizing damage. Trypan blue staining results **(**Figs. 4a-b**)** revealed blue microchannels at the microneedle puncture sites, uniformly distributed across the array area, confirming that each microneedle effectively penetrated the skin and breached the stratum corneum barrier, creating channels for drug permeation.

#### 3.2.3 Swelling behavior analysis of PIO conductive microneedles

The swelling behavior of the microneedles was assessed in vivo and in vitro to determine their performance in different environments **(Figs. S3-S4;** Table 2, Table 3**)**. In vivo, the microneedle height increased gradually from an initial value of 794 to 873 μm in 60 min, yielding a swelling ratio of 9.95 % and a slower swelling rate, which is favorable for sustained drug release. In vitro, the microneedle height rapidly increased to 1565 μm within 60 s in PBS solution, reaching a swelling ratio of 93.41 %, indicating suitability for applications requiring rapid release. The differences in swelling behavior reflect how environmental moisture variations impact microneedle usability.

**Table 2:** In vivo swelling rate of microneedles.(*n* = 3).

Time (min)	Microneedle Height (μm)	Swelling Rate (%)
0	794.0 ± 3.0	0
5	798.0 ± 4.2	0
10	820.0 ± 3.5	3.28
30	857.0 ± 4.0	7.94
45	868.0 ± 5.4	9.32
60	873.0 ± 6.1	9.95

**Table 3:** In vitro swelling rate of microneedles.(n = 3).

Time (s)	Microneedle Height (μm)	Swelling Rate (%)
0	809.0 ± 3.0	0
10	908.0 ± 4.2	12.26
20	967.0 ± 5.6	19.55
30	1235.0 ± 5.3	52.63
45	1326.0 ± 7.1	63.72
60	1565.0 ± 8.8	93.41

Compared with degradable or soluble microneedles such as those made of PLA, PVA, or HA, the P(MVE-alt-MAH) hydrogel-based microneedles used in this study exhibit rapid swelling behavior in hydrophilic environments. This property effectively prolongs the duration of ionic channel expansion in the skin, thereby facilitating more efficient ion transport. Consequently, the enlarged ionic pathways significantly enhance electrical conductivity(Liu et al., 2021).

#### 3.2.4 Conductivity evaluation

The conductivity performance of the microneedles was evaluated to determine their suitability for iontophoresis **(**Table 4**)**. The blank microneedle solution exhibited a current of 2.99 ± 0.36 mA, whereas unswollen microneedles exhibited a current of 0.08 ± 0.02 mA. After swelling, the microneedle current increased to 1.75 ± 0.43 mA, significantly improving conductivity. The swelling process increased the mobility of ionic carriers in the material, resulting in better conductivity performance and meeting iontophoresis standards.

**Table 4:** Conductivity of PIO microneedles and their matrix solution (n = 3).

Sample	Circuit Current (mA)
Blank Microneedle Solution	2.99 ± 0.36
Microneedles (Before Swelling)	0.08 ± 0.02
Microneedles (After Swelling)	1.75 ± 0.43

Furthermore, compared with carbon nanotube/graphene or ionic gel conductive systems, the hydrogel microneedles developed in this study offer significant advantages in terms of biocompatibility. This is largely attributable to their fabrication from biocompatible materials via a simplified manufacturing process(Defteralı et al., 2016). Moreover, the dissolution rate and electrical conductivity of these microneedles can be more conveniently modulated through chemical group modifications, enabling broader application potential.

#### 3.2.5 Moisture content determination of PIO conductive microneedles

The moisture content of the microneedles was measured to establish their stability and shelf life (Table S5). The moisture content of three batches of microneedles ranged from 10.54 % to 10.86 %, without significant differences between groups (*P* > 0.05), indicating a stable preparation process and consistent microneedle moisture content. Appropriate moisture content contributes to the structural integrity and mechanical strength of microneedles, extending their shelf life and ensuring their effectiveness in iontophoresis applications.

#### 3.2.6 Drug distribution uniformity

Drug distribution uniformity in the microneedles was evaluated **(Table S6)**. Drug loading in the microneedle tips was 0.7 mg, while that in the backing layer was 4.3 mg, with the drug content in the tips accounting for approximately 14 % of the total drug content. The intra-group CV% of the three microneedle groups ranged from 7.26 % to 9.07 %, all below 10 %, thereby meeting drug uniformity standards. The inter-group CV% was below 5 %, indicating good consistency in drug loading across different batches of microneedles. Uniform drug distribution ensures consistent delivery doses for each microneedle patch, improving the stability and safety of therapeutic effects.

This study comprehensively characterized PIO conductive microneedles, demonstrating their superior performance in dissolution properties, morphology, skin penetration, swelling behavior, conductivity, water content, and drug distribution uniformity. TEM and FTIR analyses confirmed the homogeneous dispersion of PIO within the matrix and the formation of stable complexes. Morphological characterization revealed sharp tips and a regular arrangement, which, corroborated by histological staining, validated efficient and minimally invasive skin penetration. The contrasting in vivo and in vitro swelling behaviors highlight the microneedles' environmental adaptability and potential for controlled or rapid release. Significantly enhanced conductivity upon swelling suggests promising applications in electroporation-based drug delivery. Stable water content ensures both mechanical integrity and storage stability, while uniform drug distribution guarantees consistent dosage and enhances therapeutic reliability.

### 3.3 Effect of iontophoresis parameters on PIO conductive microneedles

#### 3.3.1 Investigation of parameters affecting iontophoresis

**Fig. 5:**
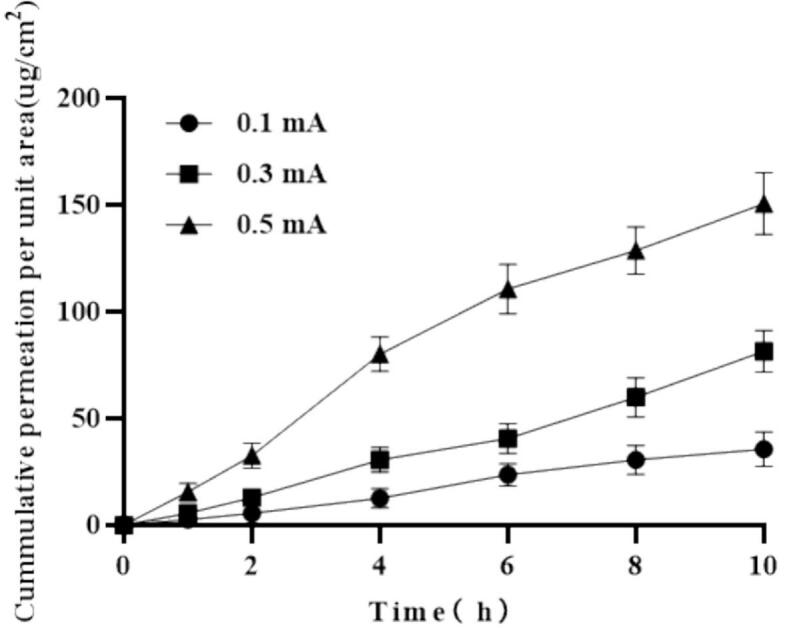
Cumulative permeability of microneedles with different currents (*n* = 4).

To investigate the effect of current intensity on drug transdermal permeation in the PIO conductive microneedle combined with iontophoresis, the cumulative permeation of the drug was evaluated under different current intensities **(**Fig. 5**)**. Over 10 h, when the current intensity was 0.5 mA, the cumulative permeation per unit area reached 150.56 ± 16.81 μg/cm^2^, while at 0.1 and 0.3 mA, the cumulative permeation was 35.56 ± 9.19 and 81.45 ± 11.14 μg/cm^2^, respectively. These findings indicate a significant positive correlation between current intensity and drug transdermal permeation (*P* < 0.05).

The increase in current intensity significantly enhanced the transdermal permeation ability of the drug. This observation is consistent with contemporary trends in iontophoresis-enhanced microneedle transdermal delivery research, which emphasize rapid improvement of transport efficiency over short time periods. These results are also in accordance with previous investigations, which have demonstrated that an iontophoretic current density of 0.5 mA/cm^2^ can substantially augment the permeation flux of specific cationic drug formulations administered via microneedle arrays(Junaid and Banga, 2022). The mechanism is attributed to higher current intensity increasing the electric field strength, providing a stronger driving force and promoting faster migration of drug ions, ultimately resulting in greater cumulative permeation. Nevertheless, excessively high currents may cause skin irritation or discomfort and even damage the microneedle structure. Hence, for safety in practical applications, a current density up to 0.5 mA/cm^2^ is recommended.

**Fig. 6:**
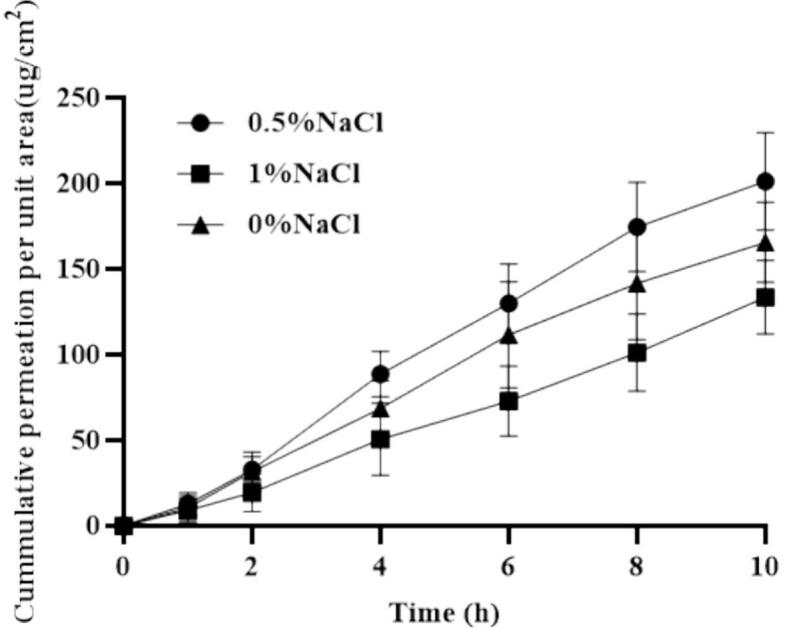
Cumulative permeability of microneedles under different NaCl concentrations (*n* = 4).

To investigate the effect of NaCl concentration on drug transdermal permeation, cumulative permeation was examined at varying NaCl concentrations **(**Fig. 6**)**. At 0.5 % (*w*/w) NaCl, the highest cumulative permeation was observed within 10 h, reaching 201.15 ± 28.38 μg/cm^2^. Conversely, permeation at 0 % and 1.0 % (*w*/w) was lower, with values of 165.59 ± 23.38 and 133.52 ± 21.54 μg/cm^2^, respectively. The results revealed that NaCl concentration exerted a significant impact on drug transdermal permeation (*P* < 0.05).

An appropriate NaCl concentration (0.5 % w/w) enhances conductivity and promotes the migration of cationic species. Both Na + and PIO are positively charged, and Na + contributes to electroosmotic flow, thereby facilitating PIO permeation. However, excessively high NaCl concentrations (e.g., 1.0 % w/w) may lead to competition between Na + and PIO for transdermal channels, ultimately reducing drug permeation. Furthermore, previous studies have indicated that the transdermal absorption of cationic drugs can be further enhanced by modulating salt ion concentrations(Wang et al., 2021). These findings are consistent with our observations, underscoring that selecting appropriate conductive conditions exerts a significant positive effect on the percutaneous absorption of PIO.

To evaluate the effect of pH on drug ionization, stability, and permeability within the microneedle system, drug transdermal permeation and formulation stability were investigated at pH values of 5.0, 6.0, and 7.0 **(Fig. S2)**. Results indicated that as the pH value increased, the drug precipitated gradually in the microneedle solution, which was most evident at pH 7.0. This suggests that higher pH values reduce drug solubility and system stability.

PIO ionization is influenced by pH. Lower pH values (5.0–6.0) favor drug ionization and solubility, facilitating uniform distribution and effective permeation within the microneedle system. Moreover, the P(MVE-alt-MAH) copolymer may undergo hydrolysis under high pH conditions, which can compromise its physical properties and encapsulation ability. Consequently, maintaining a pH between 5.0 and 6.0 is recommended to optimize drug ionization, permeation, and formulation stability.

**Fig. 7:**
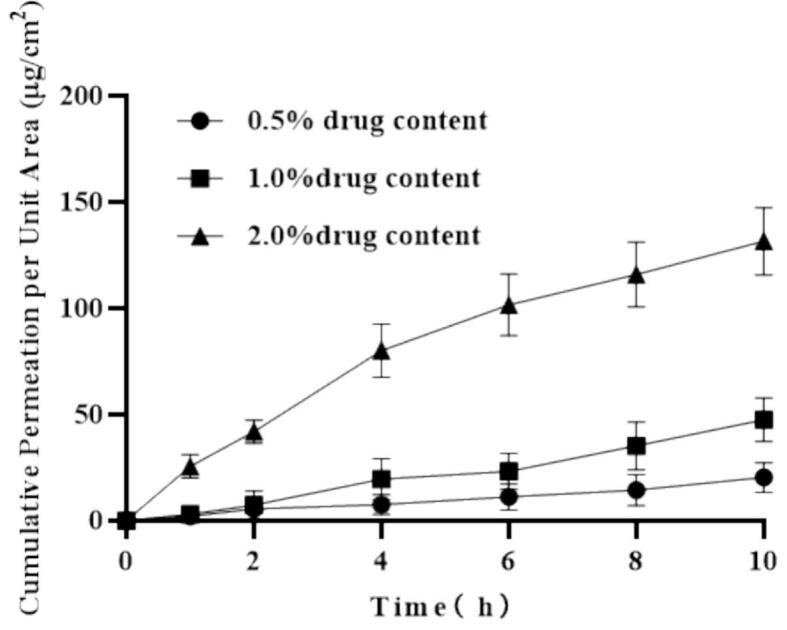
Cumulative permeability of microneedles under different drug loading concentrations (n = 4).

To investigate the effect of drug concentration on transdermal permeation, cumulative permeation was measured under drug loading concentrations of 0.5 %, 1.0 %, and 2.0 % (w/w) **(**Fig. 7**)**. The results indicated a significant increase in cumulative permeation with increased drug loading concentrations (P < 0.05). When the drug loading concentration was 2.0 % (w/w), the cumulative permeation reached 131.73 ± 17.75 μg/cm^2^ within 10 h.

Drug concentration plays a crucial role in transdermal drug delivery systems. Higher drug concentrations enhance transdermal permeation efficiency through two mechanisms: increased molecular availability for electromigration and a stronger concentration gradient driving force. However, there exists an optimal concentration threshold. Exceeding this threshold may lead to drug precipitation as concentrations approach solubility limits, compromising formulation stability. Additionally, elevated drug concentrations can result in increased viscosity, potentially affecting the mechanical properties and penetration capabilities of microneedles. Our results demonstrate that optimizing drug concentration is essential to achieve maximum permeation efficiency while maintaining both formulation stability and microneedle structural integrity.

#### 3.3.2 Effect of iontophoresis on drug transdermal permeation

**Fig. 8:**
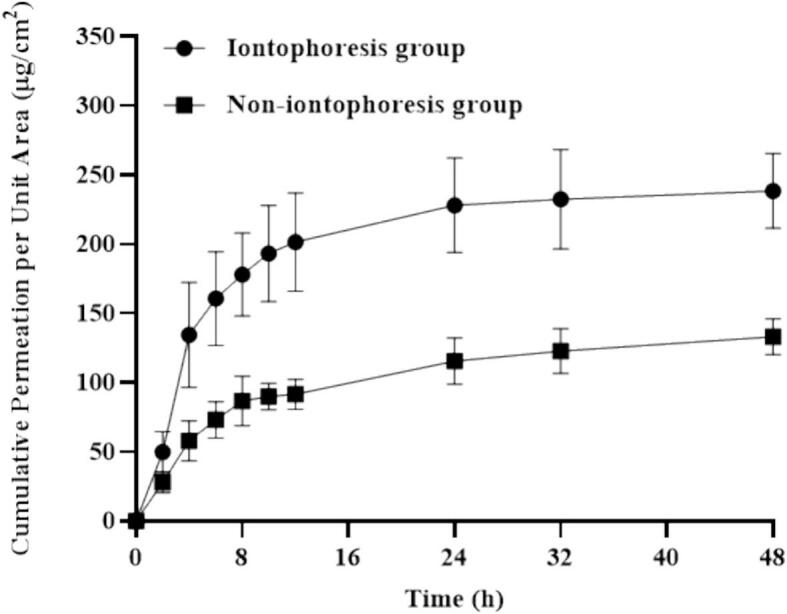
Cumulative permeability of ordinary microneedles and microneedles combined with iontophoresis (*n* = 4).

To evaluate the enhancing effect of iontophoresis on drug transdermal permeation, in vitro permeation characteristics of PIO with and without iontophoresis were compared **(**Fig. 8**)**. The cumulative permeation of the microneedle-only group tended to stabilize after 12 h, with an average permeation of 91.56 ± 10.95 μg/cm^2^ at 48 h. Conversely, the cumulative permeation of the combined iontophoresis group continued increasing within 48 h, reaching 238.1 ± 27.14 μg/cm^2^—a significant increase over the microneedle-only group (*P* < 0.001).

Iontophoresis significantly increased the migration and total permeation of the drug through electric field-driven transport. Since PIO is cationic, it can penetrate the skin barrier more effectively under the influence of an electric field. Moreover, electroosmotic flow may facilitate the release of the drug from the microneedle backing layer. The initial drug loading in the backing layer was 4.3 mg, while the drug loading in the microneedle tips was 0.7 mg. These indicate that iontophoresis can significantly enhance the transdermal delivery efficiency of the microneedle system.

The results demonstrated that ion electroporation significantly enhances the transdermal delivery efficiency of PIO via microneedles. Compared to the microneedle-only group, the electroporation-combined group exhibited an approximately 2.6-fold increase in cumulative drug permeation over 48 h (P < 0.001), highlighting the efficacy of electric field-driven drug delivery. This enhancement is attributed to the direct electromigration of the cationic drug PIO and the electroosmotic facilitation of drug release, potentially accompanied by alterations in skin conductivity and permeability. In contrast to passive diffusion, electroporation achieved faster and higher total drug delivery, which is particularly relevant for clinical applications requiring rapid and efficient drug administration. However, this study suggests the need for further in vivo validation to achieve optimal delivery outcomes. In summary, the integration of electroporation with microneedles demonstrates substantial potential for improving PIO transdermal delivery, providing a significant foundation for the development of efficient transdermal drug delivery systems.

**Fig. 9:**
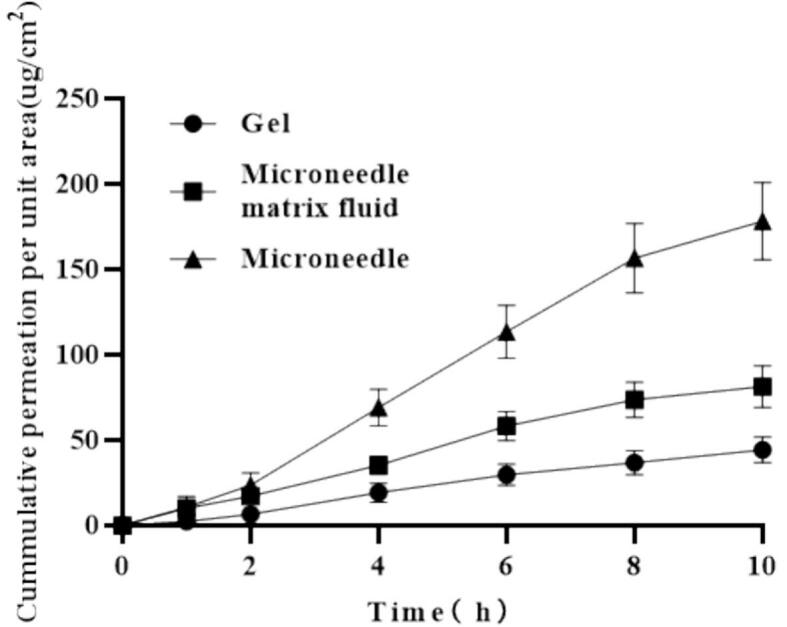
The cumulative permeability of the dosage form affects in vitro permeation.

Moreover, to investigate how dosage form influences in vitro permeation, we compared three formulations under iontophoresis: a hydrogel, a microneedle matrix solution, and fully formed microneedles **(**Fig. 9**)**. The gel exhibited a gradual rise in cumulative permeation over 10 h with 44.29 ± 7.59 μg/cm^2^, presumably driven by the electric field facilitating the migration of charged drug ions across the stratum corneum. By contrast, the microneedle matrix solution demonstrated a faster initial permeation rate, reaching 81.32 ± 12.33 μg/cm^2^ at 10 h, although increasing drug concentration in the superficial skin layers could temper further flux. In comparison, the microneedles themselves physically disrupted the stratum corneum, allowing near-instant drug penetration into deeper skin strata; combined with iontophoresis, this strategy delivered the highest 10 h cumulative permeation with 178.26 ± 22.75 μg/cm^2^ (*P* < 0.05), with negligible lag time. These differences likely stem from the gel's three-dimensional network, which functions as a sustained-release reservoir yet partially restricts diffusion, whereas the microneedle matrix solution diffuses freely but is susceptible to surface environment factors. Ultimately, the integrated microneedle platform capitalizes on physical barrier disruption and electrical driving forces to achieve rapid, deep drug transport, underscoring its potential for delivering high-molecular-weight or poorly soluble agents.

#### 3.3.3 Effects of iontophoresis on in vivo drug diffusion characteristics

**Fig. 10:**
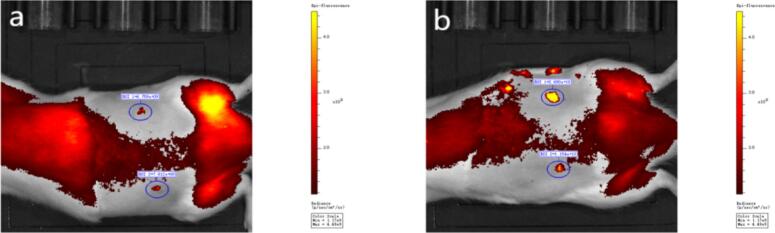
In vivo imaging results of rat skin penetration: a. Iontophoresis group (ROI1) and control group (ROI2) at 3 min post-administration; b. Iontophoresis group (ROI1) and control group (ROI2) at 30 min post-administration.

To evaluate the effects of iontophoresis on drug in vivo diffusion in the PIO conductive microneedle system, rhodamine B was used as a fluorescent marker, and IVIS technology was used to assess drug penetration and distribution in rat skin under the influence of iontophoresis **(**Fig. 10**)**. At 3 min post-administration, no significant difference was observed in fluorescence intensity between the microneedle with iontophoresis and the microneedle-only groups. However, at 30 min, the fluorescence intensity of the experimental group was significantly higher than that of the control group, reaching 2.69 × 1010 and 1.15 × 1010, respectively, with the ratio of the fluorescence intensity at 30 min to that at 3 min (Growth Factor) of 4.0-fold for the experimental group and 1.5-fold for the control group (*P* < 0.001, Table 5).

**Table 5:** Quantitative analysis results of rhodamine B in vivo fluorescence imaging.

Group	Fluorescence Intensity at 3 min (× 10^9^)	Fluorescence Intensity at 30 min (× 10^10^)	Growth Factor	*P*-value
Control group	7.81 ± 0.45	1.15 ± 0.10	1.5-fold	< 0.001
Iontophoresis group	6.76 ± 0.50	2.69 ± 0.32	4.0-fold	< 0.001

Recent research has increasingly focused on integrating fluorescence tracking methodologies with IVIS imaging systems to elucidate the diffusion dynamics and spatial distribution of drugs during combined iontophoretic and microneedle-mediated delivery(Chen et al., 2018). Empirical evidence indicates that incorporating fluorescent probes into microchannel or microneedle technologies markedly enhances both the distribution area and penetration depth of these probes within subcutaneous tissues. Moreover, higher electric field intensities have been shown to significantly accelerate the spatial distribution kinetics of fluorescent markers(Abbasi and Heath, 2024). These findings corroborate the notion that iontophoresis can notably enhance drug penetration and distribution in vivo over a relatively short timeframe. Additionally, iontophoresis may facilitate the sustained release of drugs from microneedle systems, thus improving overall delivery efficiency.

Iontophoresis parameters such as current intensity, NaCl concentration, pH value, and drug concentration have a significant impact on the transdermal permeation performance of the PIO conductive microneedle system. Optimizing these parameters can significantly enhance the transdermal permeation efficiency of the drug while ensuring system stability and safety.

### 3.4 Pharmacodynamic evaluation of PIO conductive microneedles

#### 3.4.1 Hypoglycemic pharmacodynamics of PIO

**Fig. 11:**
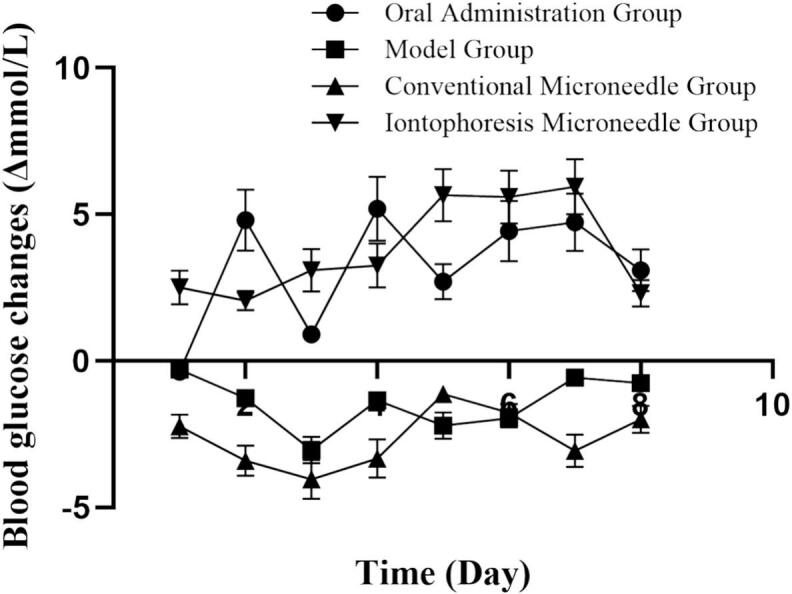
ΔBlood glucose levels of type II diabetes rats in each group for 8 consecutive days (*n* = 6).

**Fig. 12:**
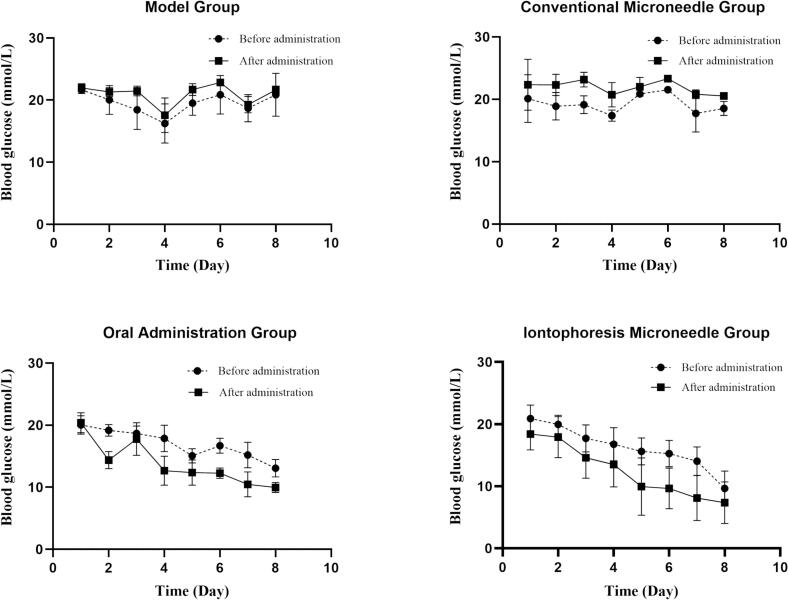
Fasting blood glucose levels of type II diabetes rats in each group for 8 consecutive days (n = 6).

To evaluate the therapeutic efficacy of the PIO conductive microneedle combined with iontophoresis for diabetes treatment, we measured and analyzed the blood glucose changes of rats in each group over 8 consecutive days **(**Fig. 11, Fig. 12**)**. The results revealed that the Δ values of the oral administration and the iontophoresis microneedle groups were greater than 0, indicating a decreasing trend in blood glucose levels. Conversely, the Δ values of the conventional microneedle and the model groups were less than 0, indicating an increasing trend in blood glucose levels. Analysis of variance revealed no significant difference in the overall hypoglycemic effects between the iontophoresis microneedle and oral control groups. Throughout the administration period, the experimental group demonstrated a continuous decrease in blood glucose levels, with fewer daily fluctuations compared with the oral control group. This implies a correlation with the sustained-release effect of the microneedle system, which ensured stable drug release and sustained therapeutic efficacy.

Although the positive control group exhibited a good hypoglycemic effect during the first 5 days after administration, blood glucose fluctuations were relatively large. Conversely, the PIO conductive microneedle combined with iontophoresis demonstrated a more desirable hypoglycemic effect, characterized by controlled blood glucose levels and reduced fluctuations. This outcome may be attributed to the sustained and steady blood drug concentration provided by the microneedle–iontophoresis system.

Several studies focusing on the transdermal delivery of hypoglycemic agents have reported significant fluctuations in blood glucose levels during glycemic control(Wang et al., 2023b). In contrast, our study demonstrates notable advantages in maintaining glycemic stability, characterized by a prolonged therapeutic duration and reduced glucose variability. Notably, iontophoresis enables a rapid elevation of systemic PIO concentrations, while the microneedle-mediated sustained-release mechanism ensures a prolonged hypoglycemic effect. This combined strategy potentially reduces dosing frequency and may offer a viable solution to address the issue of blood glucose fluctuations observed in other transdermal regimens.

The microneedle combined with iontophoresis offers several advantages in the treatment of diabetes. First, it ensures stable and efficient drug delivery, mitigating the uneven absorption and gastrointestinal discomfort associated with oral administration. Second, iontophoretic driving allows for precise control over the drug delivery rate and dose, reducing blood glucose fluctuations and improving treatment continuity and effectiveness. Third, it is painless and convenient, enhancing patient compliance. Overall, this system is promising for clinical application.

#### 3.4.2 Skin irritation of PIO conductive microneedles

**Fig. 13:**
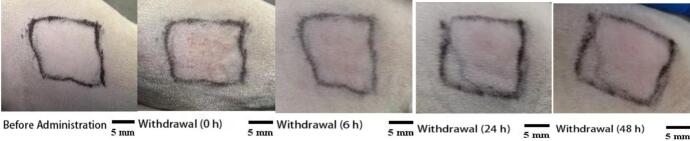
Skin irritation observation results: From left to right, images depict skin condition before administration at 0, 6, 24, and 48 h after patch removal.

To evaluate the skin safety of the PIO conductive microneedle combined with iontophoresis, irritation tests were performed on rat skin before and after administration. This included skin irritation scoring, visual observations, and histological analysis. As presented in **Table S7** and Fig. 13, mild erythema and edema (score of 1) were observed immediately after administration. The erythema and edema significantly reduced 6 h after patch removal, and the skin returned to normal 24 h later with no obvious irritation reactions.

**Fig. 14:**
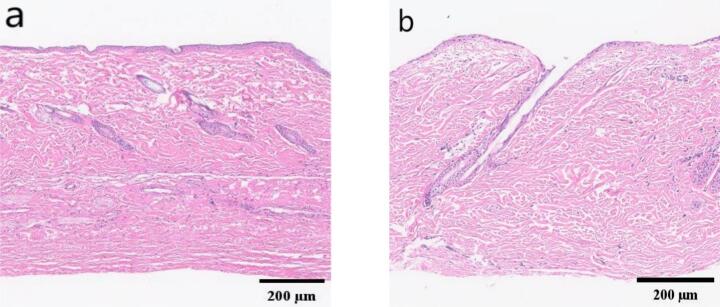
Rat skin tissue sections at the microneedle administration site: a. Normal skin tissue; b. Skin tissue at the administration site.

Histological analysis **(**Fig. 14**)** revealed that after 8 consecutive days of administration, the skin tissue at the administration site **(**Fig. 14b**)** did not exhibit significant inflammatory cell infiltration, necrosis, or other pathological changes compared with normal skin tissue **(**Fig. 14a**)**, indicating that the microneedle system did not cause significant damage to the skin tissue and possessed good biocompatibility.

Combining the results of skin irritation scoring, visual observation, and histological analysis, the PIO conductive microneedle combined with iontophoresis caused only mild, transient skin irritation during administration, with full recovery occurring within 24 h. This suggests that the system exhibits high skin safety and tolerability, making it suitable for long-term or repeated therapeutic applications.

Furthermore, this study demonstrated that the PIO conductive microneedle combined with iontophoresis exhibited excellent hypoglycemic effects in a type II diabetic rat model, significantly outperforming traditional oral administration methods. By enhancing the transdermal permeation ability of the drug, this system achieved stable and efficient blood glucose control while maintaining good tolerability in terms of skin safety, indicating its potential value for clinical application.

### 3.5 Pharmacokinetic evaluation of PIO conductive microneedles

#### 3.5.1 HPLC-MS/MS method validation

The approach met the criteria for pharmacokinetic studies in terms of specificity, linearity, accuracy, and precision.

Under the optimized conditions, PIO and IS exhibited distinct retention times with no overlapping peaks or additional degradation peaks. And the calibration curves were constructed by spiking plasma with serial concentrations of PIO (2–1000 ng/mL) and processing each sample identically. A correlation coefficient of 0.999 was achieved over this range, and the LLOQ was 2 ng/mL with a signal-to-noise ratio of approximately 10. The accuracy at the LLOQ was 90.4 %, falling within the acceptable 80–120 % range. Concentrations above the LLOQ (2–1000 ng/mL) also showed acceptable accuracies between 85 and 115 %.

Intra- and inter-day precision and accuracy were assessed at four QC levels (2, 5, 150, and 700 ng/mL). Each concentration was measured in triplicate across three batches. The RSD were below 3.0 %, and accuracies were within 85–115 %, indicating good reproducibility.

Recoveries were evaluated by comparing peak areas of PIO in plasma QC samples before and after protein precipitation with corresponding neat standard solutions. Matrix effects were calculated similarly by analyzing blank plasma extracts spiked post-extraction versus neat standards. All values were within the acceptable range (≥ 90 %), and no significant matrix effect (< 10 %) was observed.

QC samples at low, medium, and high concentrations were stable at room temperature for up to 12 h, after three freeze-thaw cycles, and during 15 days of storage at −20 °C (RE < 15 %). No substantial carryover was detected when injecting a blank sample immediately after a high-concentration QC.

Overall, this HPLC-MS/MS method meets the FDA bioanalytical guidelines for specificity, linearity, accuracy, precision, and sensitivity. It is therefore suitable for quantifying PIO plasma concentrations in the subsequent pharmacokinetic studies.

#### 3.5.2 Pharmacokinetic chrarcteristics of PIO conductive microneedles

To evaluate the pharmacokinetic characteristics of the PIO conductive microneedles combined with iontophoresis and compare them with the traditional oral administration method, plasma drug concentration-time curves and related pharmacokinetic parameters were determined and analyzed for each group of rats. The high-, medium-, and low-dose groups received full microneedle patches at doses of 30, 20, and 10 mg/kg, respectively, with tip doses calculated as 14 %, yielding 4.2, 2.8, and 1.4 mg/kg. The ordinary microneedle group received a tip dose of 1.4 mg/kg, and the oral positive control group received a dose of 1.58 mg/kg.

**Fig. 15:**
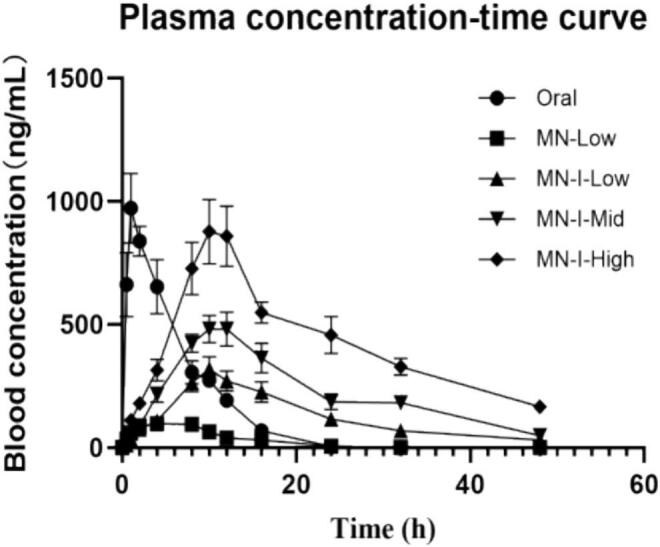
Plasma concentration-time curves of each group (n = 6).

Table 6**and**Fig. 15 illustrates the plasma concentration-time curves for each administration group. The high-dose experimental group (MN-I-High) demonstrated a Cmax of 940.03 ± 117.13 ng/mL, slightly lower than that of the oral control group (983.20 ± 128.94 ng/mL); however, the difference was not statistically significant (*P* > 0.05). The high-dose experimental group demonstrated an AUC0–∞ of 24,017.39 ± 1362.67 h·ng/mL, significantly higher than that of the oral control group (6758.03 ± 521.31 h·ng/mL), approximately 3.56 times higher. This suggests that iontophoresis may play a key role in drug release from the backing layer, which contains a higher drug content and can continuously release the drug through iontophoresis under the influence of an electric field, resulting in prolonged drug absorption.

**Table 6:** Pharmacokinetic parameters of each group (mean ± S.D., *n* = 6).

Group	Oral Control	MN-I-LOW	MN-I-MID	MN-I-HIGH	MN- LOW
AUC_0-inf_	6758.03 ± 521.31	6559.86 ± 264.90	11,563.82 ± 593.22	24,017.39 ± 1362.67	1249.74 ± 94.98
AUC_0-last_	6744.35 ± 520.08	6052.40 ± 239.01	10,740.63 ± 629.42	20,027.65 ± 977.88	1213.14 ± 90.54
C_last_	4.32 ± 0.63	30.90 ± 4.94	48.66 ± 5.47	166.03 ± 17.62	5.98 ± 1.04
C_max_	983.20 ± 128.94	329.60 ± 47.35	522.47 ± 33.29	940.03 ± 117.13	108.14 ± 9.04
T_1/2_	2.18 ± 0.14	11.31 ± 0.78	11.67 ± 1.04	16.61 ± 1.69	4.21 ± 0.40
MRT_0-inf_	5.68 ± 0.15	21.14 ± 1.13	21.87 ± 0.68	28.63 ± 1.60	8.82 ± 0.18
T_max_	1.33 ± 0.52	6559.86 ± 264.90	11.33 ± 1.03	10.33 ± 1.51	6.00 ± 2.19

In addition, The high-dose experimental group demonstrated a T_max_ of 10.33 ± 1.51 h, significantly 1·1 higher than that of the oral control group (1.33 ± 0.52 h, *P* < 0.05), indicating that the microneedle combined with iontophoresis can achieve sustained drug release and prolong the duration of action. Additionally, the high-dose experimental group displayed a significantly longer T_1/2_ value of 16.61 ± 1.69 h compared with the oral control group (2.18 ± 0.14 h, P < 0.05), indicating an extended drug residence time in the body. This is beneficial for maintaining stable plasma drug concentrations. Furthermore, the high-dose experimental group exhibited a significantly higher MRT_0–∞_ of 28.63 ± 1.60 h compared with the oral control group (5.68 ± 0.15 h, P < 0.05), further supporting the sustained-release characteristics of the drug delivery system.

Pharmacokinetic parameters were compared between groups using ANOVA and post hoc tests. The high-dose experimental group demonstrated a significantly higher AUC_0–∞_ than the oral control group (P < 0.05), indicating an increase in total drug exposure and sufficient absorption. The high-dose experimental group exhibited a significantly higher C_max_ than the control group (P < 0.05); however, no statistically significant difference was observed compared with the positive control group (P > 0.05). The high-dose experimental group displayed a significantly longer T_max_ (P < 0.05), demonstrating sustained release. The high-dose experimental group demonstrated significantly higher T_1/2_ and MRT_0–∞_ compared with the oral control group (P < 0.05), indicating a longer drug maintenance time in the body, which is beneficial for maintaining stable plasma drug concentrations.

Multiple studies have documented that microneedle delivery systems can significantly alter pharmacokinetic parameters such as AUC, Tmax, and MRT, with further prolonged drug retention achieved through synergistic application of electrode, ultrasound, or thermal enhancement technologies(Zafar et al., 2022). Our findings corroborate this trend: the combination of iontophoresis-driven delivery and sustained release from the backing layer led to significantly enhanced AUC and MRT values.

However, we also observed that in the absence of iontophoresis, transdermal absorption was slower, and Cmax was relatively lower. This may fail to achieve the desired hypoglycemic effect. Iontophoresis partially compensated for this shortcoming by accelerating drug penetration via the electric field, increasing the peak concentration and total exposure of the drug.Furthermore, the hydrogel microneedles used in this study were removed after 8 h of application. We discovered that the drug had not been completely released from the microneedles, resulting in incomplete drug absorption and utilization. This phenomenon differs from soluble microneedles, which can leave the drug in the skin, ensuring the complete release and absorption of the drug. Therefore, the administration time and release characteristics of hydrogel microneedles must be further optimized to ensure complete drug release and maximum absorption and utilization.

The PIO conductive microneedle combined with iontophoresis outperformed traditional oral administration in terms of pharmacokinetics. This system enhanced transdermal absorption and provided sustained release, resulting in more efficient and stable blood glucose control, highlighting its significant clinical application potential. Future studies should focus on optimizing administration parameters, evaluating long-term safety and efficacy, and providing evidence for feasibility in human clinical applications.

## 4 Conclusion

In this study, a novel conductive hydrogel microneedle system combined with iontophoresis was successfully developed to improve PIO transdermal delivery for the treatment of type II diabetes. By using P(MVE-alt-MAH) as the primary matrix material, we created a microneedle formulation with superior conductivity, high drug loading capacity, and excellent mechanical properties. The microneedles demonstrated effective skin penetration and provided controlled drug release.

Optimization studies of iontophoresis parameters revealed that factors such as current intensity, NaCl concentration, pH value, and drug concentration significantly influenced PIO transdermal permeation. The combination of microneedles with iontophoresis significantly increased drug permeation compared with using microneedles alone, indicating a synergistic effect.

In vivo pharmacodynamic investigations in a type II diabetic rat model revealed that the microneedle-iontophoresis system achieved superior hypoglycemic effects than oral administration. The system provided sustained blood glucose reduction with minimal fluctuations, improving therapeutic efficacy while potentially reducing systemic side effects associated with oral pioglitazone. Skin irritation assessments confirmed the safety and biocompatibility of the system, with only mild and transient skin reactions observed.

Pharmacokinetic analysis indicated that the microneedle-iontophoresis system exhibited higher bioavailability, prolonged T_1/2_, and sustained drug release, highlighting its potential for maintaining therapeutic drug levels over time.

Moreover, with advances in miniaturization and digitization of electronic devices, the development of wearable and intelligent iontophoretic drug delivery systems combined with suitable dosage forms has emerged as a highly promising direction. These systems can not only deliver drugs precisely but can also seamlessly integrate with smart devices and health monitoring systems, allowing for real-time data collection and feedback. In the future, intelligent drug delivery systems may incorporate built-in sensors to monitor patients' physiological parameters, such as blood glucose levels and heart rate, and dynamically adjust drug release rates based on the monitoring results, enabling personalized treatment.

Overall, conductive hydrogel microneedles combined with iontophoresis provide a promising strategy for PIO transdermal delivery, addressing the limitations of oral administration. This iontophoresis-microneedle drug delivery system holds significant potential for diabetes management and marks a significant advancement in transdermal drug delivery technology.

## Funding

This reasearch was financially supported by the Joint Funds of the 10.13039/501100004731Zhejiang Provincial Natural Science Foundation of China under Grant [No. LHDMZ22H300013], Basic Scientific Research Funds of Department of Education of Zhejiang Province, Zhejiang Provincial Basic Public Welfare Research Program [LTGD23H300001] and Scientific Research Fund of Zhejiang Provincial Education Department [Y202352530].

## Institutional review board statement

The animal study protocol was approved by the Institutional Review Board (or Ethics Committee) of institutional animal care and use committee of Zhejiang laboratory animal center (protocol code No. ZJCLA-IACUC-20020171).

## Informed consent statement

Not applicable.

## CRediT authorship contribution statement

**Jianling Hu:** Writing – review & editing, Writing – original draft, Investigation, Conceptualization. **Yue An:** Visualization, Supervision, Investigation. **Weiqing Wang:** Methodology. **Jing Yang:** Visualization. **Wenxin Niu:** Investigation. **Xiumei Jiang:** Investigation. **Kun Li:** Investigation. **Changzhao Jiang:** Writing – review & editing, Writing – original draft, Supervision, Conceptualization. **Jincui Ye:** Writing – review & editing, Writing – original draft, Supervision, Conceptualization.

## Declaration of competing interest

The authors declare no conflict of interes.

## Data Availability

Data will be made available on request.

## Appendix A Supplementary data

Supplementary material Image 1
