# Supplementary material for: Enhanced transdermal delivery of pioglitazone hydrochloride via conductive hydrogel microneedles combined with iontophoresis
Source: Int J Pharm X. 2025 Feb 10;9:100317. doi: 10.1016/j.ijpx.2025.100317 (PMC11871479; doi:10.1016/j.ijpx.2025.100317)
Supplement: Supplementary file 1 — Supplementary material [file mmc1.docx]

# Appendix A. Supplementary data

**Enhanced Transdermal Delivery of Pioglitazone Hydrochloride via Conductive Hydrogel Microneedles Combined with Iontophoresis**

Jianling Hu1,Yue An1,Weiqing Wang1,Jing Yang2,Wenxin Niu1,Xiumei Jiang1 , Kun Li1, Changzhao Jiang1,*, Jincui Ye1*.

1 Key Laboratory of Neuropsychiatric Drug Research of Zhejiang Province, Institute of Materia Medica, Hangzhou Medical College, Hangzhou, 310013, China

2 Collaborative Innovation Center of Green Pharmaceuticals, Zhejiang University of Technology, Hangzhou 310014, China

* Corresponding author: Changzhao Jiang and Jincui Ye, address: Hangzhou Medical College, Hangzhou, 310013, China, e-mail: my100203921@gmail.com (C. Jiang), yejincui@163.com (J. Ye) Corresponding author at: Institute of Materia Medica, Hangzhou Medical College,Hangzhou, 310013, China.


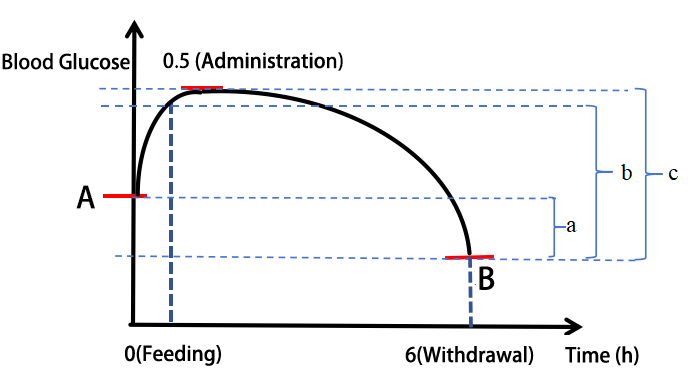


**Figure S1.** Schematic illustration of pioglitazone hydrochloride delivery via microneedle-assisted iontophoresis. The blood glucose changes were monitored through three parameters: (a) Δ glucose level (pre-feeding vs. post-administration), (b) Δ glucose level (administration time vs. post-administration), and (c) Δ maximum glucose elevation (peak vs. post-administration).


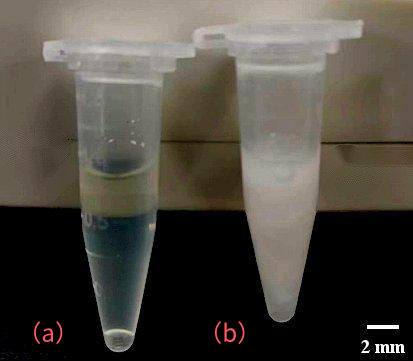


**Figure S2.** Comparison of pH regulation effects of drug-loaded microneedle solutions: a. Solution at pH 7.0; b. Solution at pH 5.0 demonstrating complete dissolution


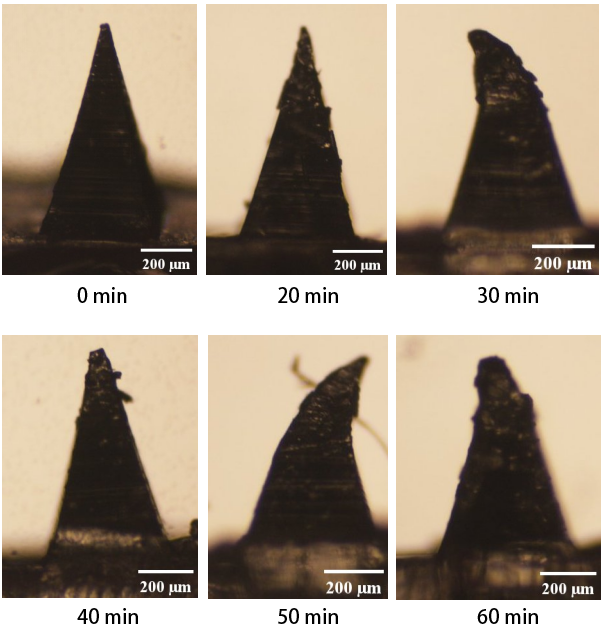


**Figure S3.** In vivo swelling characteristics of microneedles.

**
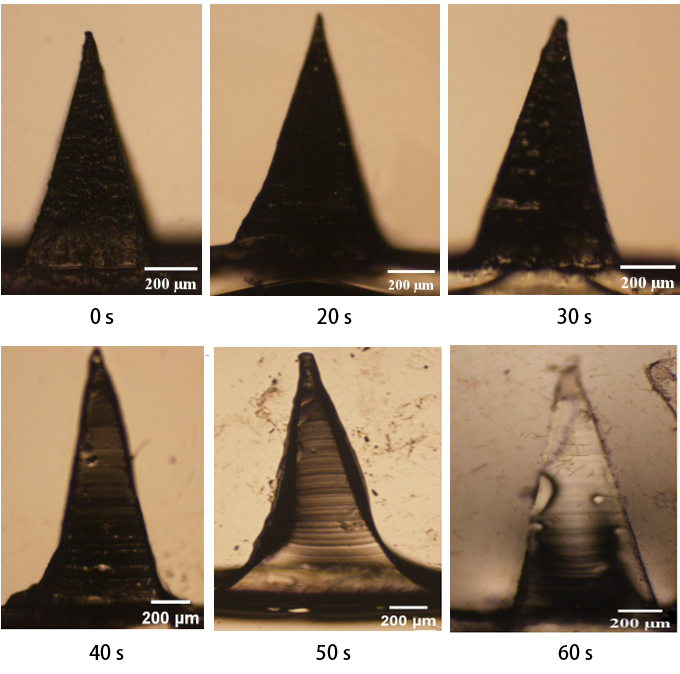
**

**Figure S4.** In vitro swelling characteristics of microneedles.

**Table S1** Matrix Material and Formulation Information

| Types of Auxiliary material | Dosage of Auxiliary material (w/v%) | Dosage of Primary Material [P(MVE-alt-MAH)](w/v%) |
| --- | --- | --- |
| HA(400 kDa) | 1.0 | 15.0 |
|  | 1.5 |  |
|  | 3.0 |  |
| PVP k90 | 1.0 | 15.0 |
|  | 1.5 |  |
|  | 3.0 |  |
| PVA 124 | 1.0 | 15.0 |
|  | 1.5 |  |
|  | 3.0 |  |
| PVA 350 | 1.0 | 15.0 |
|  | 1.5 |  |
|  | 3.0 |  |

**Table S2. Gradient elution program for HPLC analysis.**

| Time (min) | A (0.1% Formic Acid Aqueous Solution) | B (Acetonitrile) |
| --- | --- | --- |
| 0 | 90 | 10 |
| 2 | 50 | 50 |
| 4 | 10 | 90 |
| 6 | 90 | 10 |

**Table S3.** PIO solubility in various solutions (mean ± S.D.).

| Solution | Concentration (µg/mL) |
| --- | --- |
| Purified Water | 1854.55 ± 257.22 |
| Ethanol | 8876.69 ± 313.69 |
| PBS | 5.79 ± 3.64 |
| Physiological Saline | 1193.39 ± 105.85 |
| P(MVE-alt-MAH) Solution (5% w/w) | 24489.80 ± 1478.56 |

Table S4. Microneedle matrix material penetration performance test.

| Material Type | Primary Material:Auxiliary Material | Number of Parafilm Layers Penetrated | Maximum Compressive Force (N) |
| --- | --- | --- | --- |
| PVA 124 | 5:1 | 2 | N/A |
|  | 10:1 | 3 | 22 |
|  | 15:1 | 3 | 25 |
| PVA 350 | 5:1 | 2 | N/A |
|  | 10:1 | 3 | 39 |
|  | 15:1 | 3 | 16 |

**Table S5.** Moisture content determination of conductive microneedles.

| Microneedle Batch | Sample Number | Weight Before Drying (m0) | Weight After Drying (mf) | Moisture Content (%) |
| --- | --- | --- | --- | --- |
| 1 | 1 | 132.50 | 118.70 | 10.54 ± 0.14 |
|  | 2 | 131.80 | 117.70 |  |
|  | 3 | 132.30 | 118.40 |  |
| 2 | 1 | 132.30 | 118.20 | 10.55 ± 0.27 |
|  | 2 | 132.10 | 117.90 |  |
|  | 3 | 132.70 | 119.10 |  |
| 3 | 1 | 131.30 | 116.80 | 10.86 ± 0.24 |
|  | 2 | 130.60 | 116.30 |  |
|  | 3 | 131.30 | 117.40 |  |

**Table S6.** Determination of microneedle drug uniformity.

| **Microneedle Group** | **Average Drug Content per Region (µg)** | **Intra-group CV (%)** | **Inter-group CV (%)** |
| --- | --- | --- | --- |
| 1 | 505.35 ± 42.70 | 9.07% | 2.21% |
| 2 | 486.33 ± 37.20 | 7.65% |  |
| 3 | 505.51 ± 36.72 | 7.26% |  |

**Table S7.** Skin irritation scores of microneedle combined with iontophoresis system in rats.

| **Time Point** | **Erythema Score (0–3)** | **Edema Score (0–3)** | **Erosion Score (0–3)** |
| --- | --- | --- | --- |
| Before Administration | 0 | 0 | 0 |
| Immediately After Withdrawal (0 h) | 1 | 1 | 0 |
| Immediately After Withdrawal (6 h) | 0 | 0 | 0 |
| Immediately After Withdrawal (24 h) | 0 | 0 | 0 |
| Immediately After Withdrawal (48 h) | 0 | 0 | 0 |
